# An Evaluation of Plotless Sampling Using Vegetation Simulations and Field Data from a Mangrove Forest

**DOI:** 10.1371/journal.pone.0067201

**Published:** 2013-06-27

**Authors:** Renske Hijbeek, Nico Koedam, Md Nabiul Islam Khan, James Gitundu Kairo, Johan Schoukens, Farid Dahdouh-Guebas

**Affiliations:** 1 Laboratory of Plant Biology and Nature Management, Vrije Universiteit Brussel, Brussels, Belgium; 2 Plant Production Systems, Wageningen University and Research Centre, Wageningen, The Netherlands; 3 Mombasa Research Centre, Kenya Marine and Fisheries Research Institute, Mombasa, Kenya; 4 Department of Fundamental Electricity and Instrumentation, Vrije Universiteit Brussel, Brussels, Belgium; 5 Laboratory of Systems Ecology and Resource Management, Université Libre de Bruxelles, Brussels, Belgium; Cirad, France

## Abstract

In vegetation science and forest management, tree density is often used as a variable. To determine the value of this variable, reliable field methods are necessary. When vegetation is sparse or not easily accessible, the use of sample plots is not feasible in the field. Therefore, plotless methods, like the Point Centred Quarter Method, are often used as an alternative. In this study we investigate the accuracy of different plotless sampling methods. To this end, tree densities of a mangrove forest were determined and compared with estimates provided by several plotless methods. None of these methods proved accurate across all field sites with mean underestimations up to 97% and mean overestimations up to 53% in the field. Applying the methods to different vegetation patterns shows that when random spatial distributions were used the true density was included within the 95% confidence limits of all the plotless methods tested. It was also found that, besides aggregation and regularity, density trends often found in mangroves contribute to the unreliability. This outcome raises questions about the use of plotless sampling in forest monitoring and management, as well as for estimates of density-based carbon sequestration. We give recommendations to minimize errors in vegetation surveys and recommendations for further in-depth research.

## Introduction

### Mangroves

Mangroves are forests found in tropical and subtropical regions with their tree roots partly or completely in the saline substrate or surface water. They predominantly grow in intertidal areas of shorelines, exhibit a marked degree of tolerance to high salt concentrations and soil hypoxia and have propagules that are able to survive and exploit dispersal by seawater [1]. Unlike most tropical forests, mangroves have a very low species diversity [2]. Besides the internal value and beauty of mangroves, they provide a number of services [3]: i) Act as an atmospheric CO_2_ sink; ii) Are an essential source of oceanic carbon iii) Support fisheries; iv) Buffer for seagrass beds and coral reefs against the impacts of river-borne siltation v) Protect coastal communities from sea-level rise, storm surges, and tsunamis; vi) Provide essential food, fibers, timber, chemicals, and medicines for communities living close to mangroves.

High population pressure in coastal areas has led to the conversion of many mangrove areas. It has been estimated that between 1980 and 2000 the world might have lost 5 million ha of mangroves, or 25 per cent of the extent found in 1980 [4]. Numbers however, have to be taken with caution as monitoring is scarce and not comprehensive.

### Methods Available for Quantifying Biomass

Quantifying the amount of biomass in the tropics can allow for better deforestation estimates and would allow calculation of the amount of carbon lost. Currently, for most tropical forests, neither the averages nor the spatial distribution of forest biomass are known [5].

Monitoring forest stocks and forest area changes requires reliable methods. Despite the necessity, quantifying biomass in forests remains a challenge [6]. Texts on Monitoring, Reporting and Verification recommend a combination of remote sensing and ground-based forest inventory approaches [7]. In ground-based forest inventories one of the variables often used is tree density.

Determining tree density is relatively straightforward when large field plots can be laid out in which trees are counted. Alternatively, plotless sampling (sometimes spelled plot-less), also called distance methods, have been developed [8]–[11], but proven not to be reliable in all cases [12], [13]. Plotless sampling methods calculate the average area per tree by measuring distances between points and trees or between trees. These techniques have the advantage of not requiring plot boundaries and are generally fast, since inter-tree distances tend to be low in mangrove forests and therefore rapidly measured [14].

When entering a mangrove forest for research purposes, one has to be aware of the tide schedule and progress is particularly difficult when trying to get deeper into the forest as often climbing over tree roots is required. Laying plots in a mangrove forest can range from being extremely time consuming up to unfeasible. Measuring distances between trees is comparatively more easy and fast. For these reasons one plotless sampling method - the Point Centred Quarter Method (PCQM) - has been recommended for mangrove research [14] and since then been used for a variety of purposes [15]–[19]. Besides PCQM, a number of other plotless sampling methods exist.

### Research Questions

This research tries to find the accuracy of PCQM in relation to other plotless sampling methods. The objectives are threefold: i) To evaluate the accuracy of PCQM both with field and simulation data; ii) To compare the accuracy of PCQM with other plotless sampling methods; iii) To relate results obtained from field data to different vegetation patterns.

### Set-up of the Study

This study consists of two parts: i) an observation-based study and ii) a simulation-based study. In the first part, the locations of all trees were mapped in four sites in a mangrove forest. In the second part tree patterns were simulated using MATLAB TM (student version 7.7.0 R2008b The Mathworks). As such, two different types of data sets were acquired on which different plotless methods were tested. In each of the following sections, first the observation-based study and then the simulation-based study will be discussed.

## Methods

### Ethics Statement

For this study, fieldwork was conducted in a mangrove forest at Gazi bay in the Coast Province of Kenya. For this area, the Kenyan Ministry of Science and Technology issued a research clearance permit (no. NCST 5/002/R/158). Consequently, all necessary permits were obtained for the described field studies.

### Observation-based Study

To find the accuracy of a sampling method, one should first know the actual (true) density. We established the true density of four sites in a mangrove forest in Gazi bay, Kenya (4°25 S and 39°30E) by counting and mapping all trees in these four stands. This means that we could reproduce the forest stands on a computer to test the plotless sampling methods as if they were applied in the field. In plotless sampling distances are measured *between* trees and therefore the tree location is the relevant variable. We digitized all tree locations and developed an algorithm for each plotless sampling method. This allowed numerous repetitions of the density estimations and thus the calculation of the mean estimate and confidence intervals for each sampling method.

#### Mapping tree locations in the field

In the mangrove forest in Gazi bay 10 mangrove species have been reported [20]: Avicennia marina (Forssk.)Vierh., Bruguiera gymnorrhiza (L.) Lam, Ceriops tagal (Perr.) C.B. Robinson, Heritiera littoralis Dryand, Lumnitzera racemosa Willd, Rhizophora mucronata Lam, Sonneratia alba Sm, Xylocarpus granatum Koen and Xylocarpus moluccensis (Lamk.) Roem [21]. In this area mangrove zonation has been previously described in detail [22].

**Figure 1:**
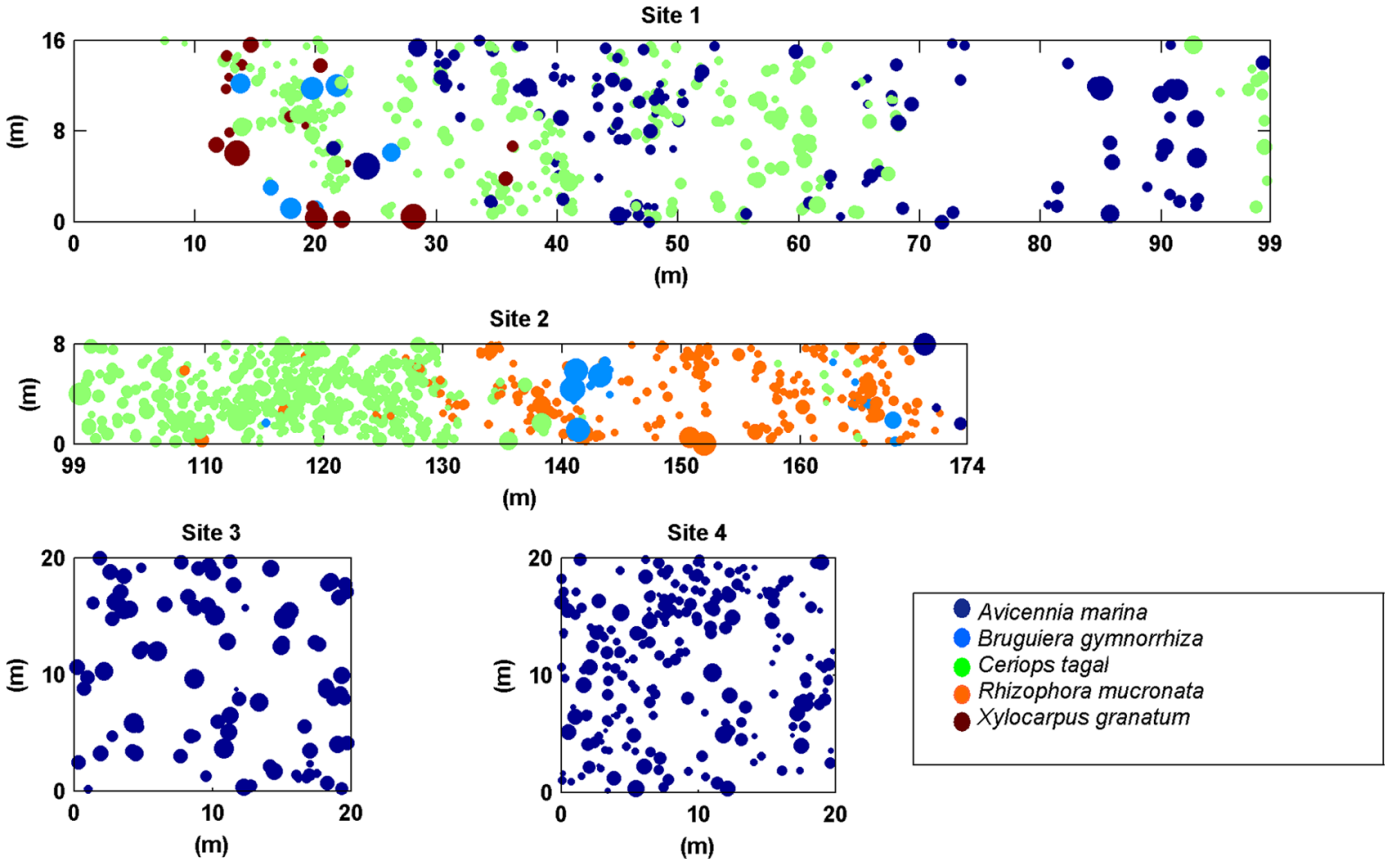
Spatial distribution of mangrove trees in 4 field sites at Gazi Bay. The dots show the location of each tree. The diameter of the dots is proportional to the diameter of the stem above the root. The colour of the dots represents the tree species: Dark blue is *Avicennia marina*, light blue is *Bruguiera gymnorrhiza*, green is *Ceriops tagal*, orange is *Rhizophora mucronata* and brown is *Xylocarpus granatum*. Together site 1 and 2 form a complete section of the forest; from low tide shore to terrestrial vegetation (supralittoral level). The y-axis of site 1 starts at the terrestrial vegetation and ends at the beginning of site 2. The y-axis of site 2 ends at the last tree at the creek side. Site 3 had closed canopy, while site 4 had open canopy.

In February and March of 2009, x- and y- coordinates of single trees were recorded in four sites at Gazi Bay. This was done by making field grids of 10 m by 10 m, 5 m by 5 m, and 2 m by 2 m and measuring the distances from the trees to the grid borders. Site 1 and 2 were located in an area with multiple mangrove species, while site 3 and 4 were in a monospecific *Avicennia marina* stand. Together, site 1 and 2 stretched from the shore to land, forming a complete section of the mangrove zonation. Site 3 was chosen in an area with closed canopy and site 4 in an area with open canopy. In site 1 and 2 all trees higher than 1.30 m were recorded, while in site 3 and 4 all trees higher than 0.50 m were recorded. Both heights represent phases of survival and target trees that are most likely to be recruited into the adult tree layer. Figure 1 shows all the coordinates of the trees in the four sites.

### Simulation-based Study

From previous studies it is known that errors in plotless sampling can be (partly) ascribed to the degree of non-randomness of a vegetation pattern. [23]. In vegetation analysis, two basic types of spatial patterns are known besides randomness: regular and aggregated dispersion, where dispersion refers to the arrangement of points in a plane [24]. In mangrove forests an additional spatial pattern exists: species show a differential distribution perpendicular to the coastline (parallel to elevation). This pattern is probably due to the different physiological adaptations and different tolerance levels to, for example, salinity, resulting in different optimal growth conditions and hence position (Saenger 2002). The pattern has been referred to as zonation, which can be discrete or gradual.

To gain more insight into how the accuracy of the methods varies with the vegetation patterns, six spatial patterns were created in MATLAB: a regular, random and aggregated pattern and each of these three with or without a distributional gradient (zonation).

First, random, aggregated and semi-regular pattern were generated: for the random pattern the MATLAB function *rand()* was used. This function draws pseudorandom values from the standard uniform distribution. The aggregated pattern was generated by clustering trees around uniform random spots. On average these clusters contained 10 trees. The trees within the aggregates had a uniform random distribution. The semi- regular pattern was generated by placing points at regular distances along the x- and y-direction in the plane. Second, a gradient (density trend) was applied to all three patterns in the y-direction with a natural logarithm, creating three new spatial patterns with zonation.

#### Tree density of the vegetation patterns

**Figure 2:**
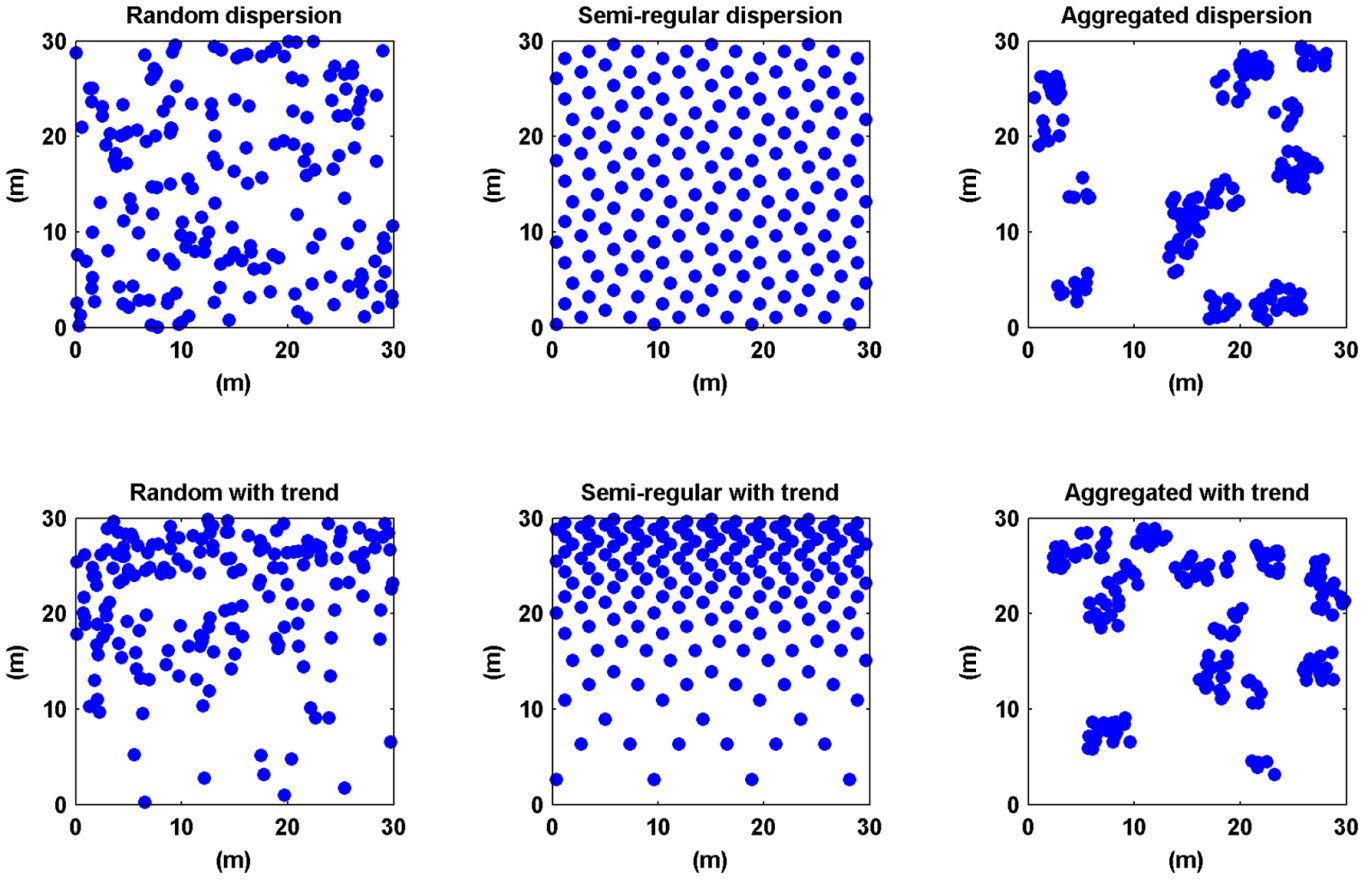
Vegetation patterns. Spatial point patterns representing 6 possible vegetation distributions (from left to right and from top to bottom): Random, semi-regular, aggregated, random with a density trend, semi-regular with a density trend and aggregated with a density trend.

In our 4 field sites, densities were found between 0.08 and 1.22 trees/m^2^. To test the effect of different vegetation patterns we used a density of 0.2 trees/m^2^ with plot sizes of 100 m by 100 m. The acquired data sets were used to test a number of plotless sampling methods. These large plot sizes allowed us to also compare higher order methods (for which the field sites were too small). A visualisation of the resulting six patterns with tree densities of 0.2 trees/m^2^ is given in figure 2.

The question is if relative errors will change if this density would vary. To find the effect of the density itself on the accuracy of the density estimations we made an additional analysis in which we varied the density of each vegetation patterns between 0.05 and 1 trees/m^2^.

### Testing Plotless Sampling Methods on the Acquired Data Sets

As far as we know, PCQM is the only plotless sampling method used in mangrove research. To investigate possible alternative techniques, this evaluation is extended with four categories of plotless sampling found in the literature: the Nearest Neighbour methods, the Basic Distance methods, the Ordered Distance methods, the Variable Area Transect methods and the Angle Order methods of which PCQM is part. In each method distances are measured between trees or between points and trees to estimate tree density. For each category a description, equation and reference can be found in table 1.

**Table 1:** An overview of the plotless sampling methods evaluated in this study.

Method	Description of distance(s) measured	Equation	Literature source
Nearest neighbour	The distance between a tree and the nearest tree is measured.	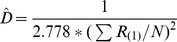	[8], [12], [13]
Basic distance	The distance between a point and the nearest tree is measured.	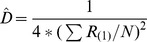	[8]
Ordered distance 1	The distance between a point and the nearest tree is measured.	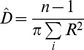	[9], [28]
Ordered distance 2	The distance between a point and the second nearest tree is measured.	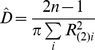	[9], [28]
Ordered distance 3	The distance between a point and the third nearest tree is measured.	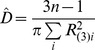	[9], [28]
PCQM 1	The distances between a point and the nearest tree in each quadrant around the point are measured.	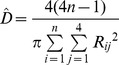	[9], [11]
PCQM 2	The distances between a point and the second nearest tree in each quadrant around the point are measured.	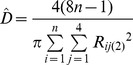	[9]
PCQM 3	The distances between a point and the third nearest tree in each quadrant around the point are measured.	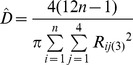	[9]
Variable Area Transect	The distance from a point to the g^th^ individual in a given direction with a certain width (a transect) is measured.	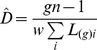	[10]

D = estimated density, R = distance measured in the field, i = number of sampling point, j = number of quadrant, g = the g^th^ individual, L is length of transect and W is width of transect, N or n = number of sampling points.

For each method an algorithm was developed using MATLAB. The algorithms were used to test the plotless sampling methods on the data sets acquired through both the field-based and the simulation study.

The algorithms can be found in File S1 and on the website of the Université Libre de Bruxelles [25].

#### Preventing edge effects

The data sets acquired (both from the field-based and simulation-based study) are enclosed and thus contain borders. When a sampling point is located close to the border of a data set, the nearest tree measured might be farther away then would be measured in the field (for example, in the field there might be a tree right at the other side of the border, causing a smaller distance measured). This effect has been described by Peter Haase [26] and called edge effects. To prevent edge effects in this study, three actions were taken:

1. The sampling points were located at a minimal distance from the site boundaries (0.05*surface area^0.5^ for sites with more than 128 trees and 0.1*surface area^0.5^ for sites with 32 to 127 trees, corresponding to at least the average distance between two trees).
2. When a method mandated that a tree across the site boundary should be used for computation, the relevant sampling point was omitted (this applied only for PCQM and the Variable Area Transect method).
3. Higher order methods were not analysed for the field data, but only for the simulation-based study, which had large areas of 100 m. by 100 m.

#### Replications

Each method was used to estimate tree density using 5, 10, 15, 20, 25 and 30 random sampling points. These calculations were repeated ten times for each field site and pattern to allow for the mean and standard deviation calculation. The standard deviation was calculated with the following equation: 
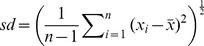
, where the mean is given by 
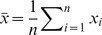
. Both the estimated mean and standard deviation did not change much above 15 sampling points. Therefore in this article only the results for estimations with 30 random sampling points are presented. The variation in mean estimation and standard deviation when using 5 to 30 sampling points can be found in File S2.

## Results

Clark and Evans [27] developed an aggregation index as a crude measure of clustering or ordering of a point pattern. A value R>1 suggest regularity, R<1 suggests clustering and R equals 1 suggests randomness. The standard range is 0<R<2.1491.To compare the degree of clustering between the field sites and the vegetation patterns, the R index was calculated for each of these.

Table 2 and 3 show the aggregation indices for both the field sites and the patterns: The patterns of the field sites vary between clustered and close to random. Sites 3 and 4 have patterns most closely approaching randomness with aggregation indices of 0.85 and 0.92 respectively. The trees in site 1 and 2 show a more diverse pattern with aggregation indices between 0.56 and 0.67. The simulated vegetation patterns show a wider range, which provides the opportunity to test the performance of plotless sampling in cases of more extreme regularity or aggregation.

**Table 2:** The aggregation indices (R) for the field sites.

Site	Aggregation index (R)
Site 1 (*Avicennia marina*)	0.67
Site 1 (*Ceriops tagal*)	0.56
Site 2 (*Ceriops tagal*)	0.59
Site 2 (*Rhizophora mucronata*)	0.64
Site 3 (*Avicennia marina*)	0.85
Site 4 (*Avicennia marina*)	0.92

**Table 3:** The aggregation indices (R) for the vegetation patterns.

Spatial pattern	Aggregation index (R)
Random	1.03
Random with gradient	0.90
Semi-regular	1.89
Semi-regular with gradient	1.46
Aggregated	0.45
Aggregated with gradient	0.42

Table 4 and 5 give an overview of the results for each method, field site and pattern. The results from the observation-based study show the error one can get when sampling in the field; the simulation-based study relates the spatial pattern to the errors.

**Table 4:** Tree density estimations using plotless sampling in four field sites (30 sampling points, 10 replicates).

Site	Exact density (n/m^2^)	NN (n/m^2^)	BD (n/m^2^)	OD1 (n/m^2^)	PCQM1 (n/m^2^)	VATX3 (n/m^2^)	VATY3 (n/m^2^)	Range of mean error (%)
Site 1 (*Avicennia marina*)	0.08	0.08	0.02	0.01	–	–	0.06	−87 to +5
*95% CI*		*0.02*	*0.01*	*0.01*	*–*	*–*	*0.01*	
Site 1 (*Ceriops tagal*)	0.20	0.20	0.02	0.01	–	–	0.09	−94 to +1
*95% CI*		*0.07*	*0.01*	*0.01*	*–*	*–*	*0.03*	
Site 2 (*Ceriops tagal*)	1.22	0.16	0.05	0.04	–	–	0.14	−97 to −87
*95% CI*		*0.10*	*0.02*	*0.02*	*–*	*–*	*0.07*	
Site 2 (*Rhizophora mucronata*)	0.49	0.11	0.11	0.07	–	–	0.14	−85 to −72
*95% CI*		*0.07*	*0.08*	*0.07*	*–*	*–*	*0.05*	
Site 3 (*Avicennia marina*)	0.22	0.33	0.18	0.19	0.15	0.21	0.21	−32 to +49
*95% CI*		*0.24*	*0.03*	*0.07*	*0.05*	*0.06*	*0.04*	
Site 4 (*Avicennia marina*)	0.57	0.50	0.50	0.43	0.36	0.47	0.46	−37 to −12
*95% CI*		*0.20*	*0.21*	*0.19*	*0.11*	*0.15*	*0.14*	
**Range of mean error (%)**		−87 to +53	−96 to −12	−97 to −13	−31 to −37	−2 to −17	−88 to −3	

Tree density estimation is the mean of 10 estimations using each method with 30 random sampling points; half 95 CI is the half width of the 95% confidence interval for the 10 estimates; NN is Nearest Neighbour; BD is Basic Distance; OD is Ordered Distance; PCQM is Point Centred Quarter Method; VAT is Variable Area Transect.

**Table 5:** Tree density estimations using plotless sampling within six vegetation patterns (30 sampling points, 10 replicates).

Spatial pattern	Exact density (n/m^2^)	NN (n/m^2^)	BD (n/m^2^)	OD1 (n/m^2^)	OD2 (n/m^2^)	OD3 (n/m^2^)	PCQM1 (n/m^2^)	PCQM2 (n/m^2^)	PCQM3 (n/m^2^)	VATX3(n/m^2^)	VATY3(n/m^2^)	Range of mean error (%)
Random	0.2	0.21	0.22	0.21	0.20	0.21	0.18	0.15	0.16	0.21	0.21	−28 to +9
*Half 95% CI*		*0.06*	*0.11*	*0.07*	*0.04*	*0.05*	*0.05*	*0.09*	*0.07*	*0.03*	*0.02*	
Random with gradient	0.2	0.06	0.07	0.04	0.04	0.05	0.03	0.04	0.04	0.06	0.07	−83 to −66
*Half 95% CI*		*0.03*	*0.04*	*0.02*	*0.02*	*0.02*	*0.02*	*0.02*	*0.02*	*0.02*	*0.03*	
Semi-regular	0.2	0.08	0.35	0.37	0.23	0.24	0.34	0.20	0.18	0.23	0.24	−61 to +84
*Half 95% CI*		*0.00*	*0.05*	*0.04*	*0.02*	*0.02*	*0.07*	*0.07*	*0.10*	*0.01*	*0.02*	
Semi-regular with gradient	0.2	0.03	0.14	0.07	0.08	0.08	0.09	0.06	0.05	0.10	0.08	−83 to −32
*Half 95% CI*		*0.02*	*0.07*	*0.03*	*0.04*	*0.03*	*0.05*	*0.03*	*0.02*	*0.02*	*0.03*	
Aggregated	0.2	0.76	0.05	0.04	0.06	0.10	0.04	0.05	0.07	0.11	0.10	−80 to +278
*Half 95% CI*		*0.32*	*0.03*	*0.02*	*0.03*	*0.03*	*0.02*	*0.03*	*0.04*	*0.03*	*0.02*	
Aggregated with gradient	0.2	0.99	0.02	0.02	0.04	0.05	0.03	0.06	0.07	0.08	0.07	−92 to +394
*Half 95% CI*		*0.47*	*0.01*	*0.01*	*0.02*	*0.02*	*0.01*	*0.03*	*0.02*	*0.04*	*0.04*	
**Range of mean error (%)**		−83 to +394	−90 to +75	−92 to +84	−81 to +16	−75 to +19	−85 to +71	−79 to +2	−80 to −11	−71 to +17	−64 to +18	

Tree density estimation is the mean of 10 estimations using each method with 30 random sampling points; half 95 CI is the half width of the 95% confidence interval for the 10 estimates; NN is Nearest Neighbour, BD is Basic Distance; OD is Ordered Distance; PCQM is Point Centred Quarter Method; VAT is Variable Area Transect.

### *Observation-based Study*

**Figure 3:**
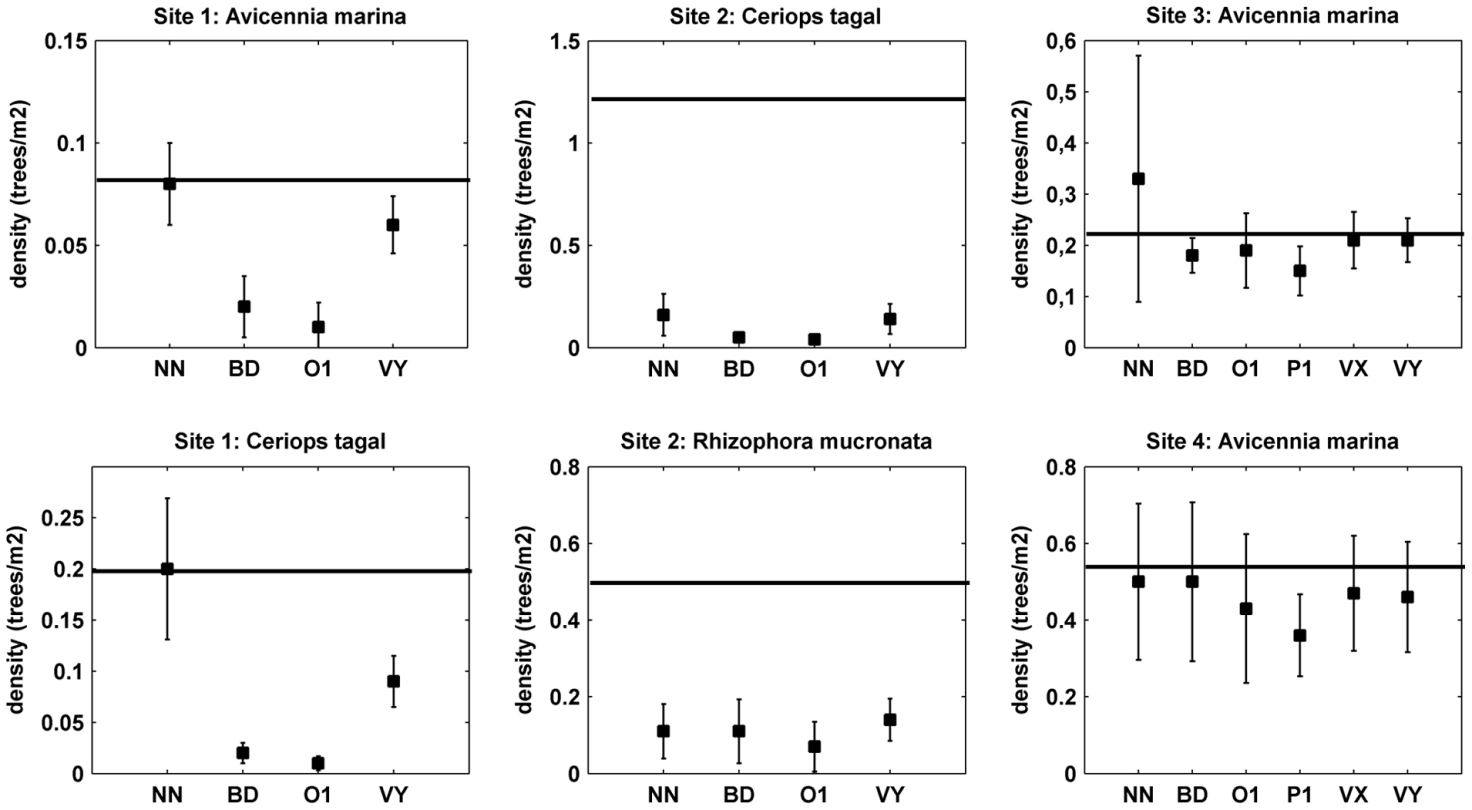
Tree density estimations by applying plotless sampling methods to field data. The density estimations found by applying plotless sampling method to the field data. The field data consisted of mangrove tree locations in four sites. In each graph the horizontal lines are the real tree densities in the sites. The closed squares are the mean of 10 estimations with 30 random sampling points. The verticals crossing the means are the 95% confidence intervals. NN = Nearest Neighbour; BD = Basic Distance; O1 = Ordered distance 1; VY = Variable Area Transect in the y-direction with 3 trees; VX = Variable Area Transect in the x-direction with 3 trees; P1 = Point Centred Quarter Method with the nearest tree in each quadrant.

In figure 3, the results for the observation-based study are shown in a graph. Using the plotless sampling methods, large underestimations were found for the density of the sites, especially in site 1 and 2. The largest underestimation (97%) was found in the estimation with the Ordered Distance Method (O1) in site 2 with the tree *Ceriops tagal*. Overall, the Variable Area Transect in the y-direction (VY) and the Nearest Neighbour (NN) method perform slightly better in the field data, but still generate errors as large as 80% in some sites.

Due to the size of the plots (see previous section about preventing edge effects), PCQM (denoted P1 in figure 3) was only tested in sites 3 and 4. In these sites, it has an underestimation of 31% and 24% respectively. These errors are larger than the errors of the other methods for these two sites.

### *Simulation-based Study*

**Figure 4:**
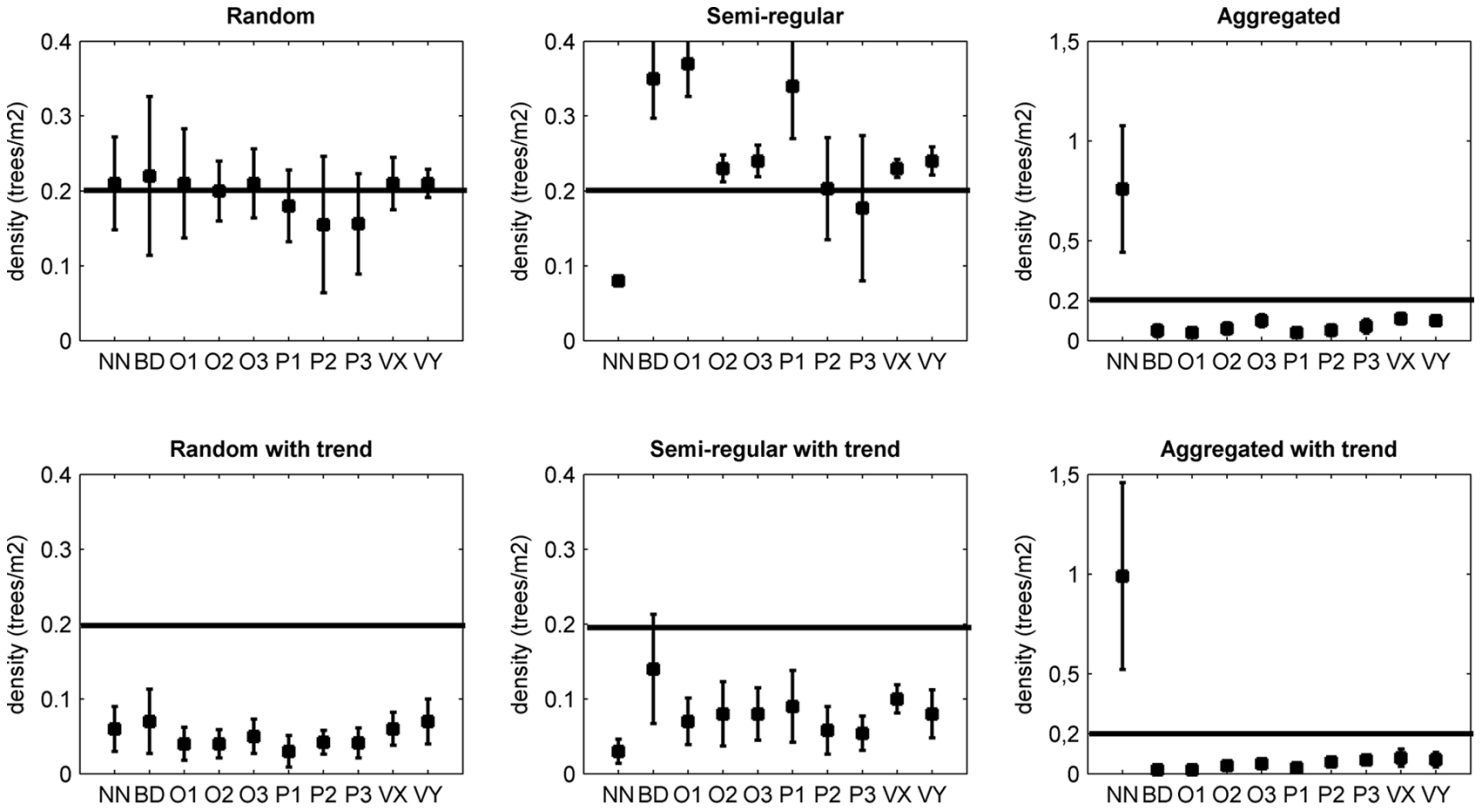
Tree density estimations by applying plotless sampling methods to 6 vegetation patterns. In each graph, the horizontal lines are the real densities (0.2 points per m^2^ for all patterns). The closed squares are the mean of 10 estimations with 30 random sampling points. The verticals crossing the means are the 95% confidence intervals. NN = Nearest Neighbour, BD = Basic Distance, O1 = Ordered distance 1, O2 = Ordered distance 2, O3 = Ordered distance 3, P1 = PCQM 1, P2 = PCQM 2, P3 = PCQM3, VX = Variable Area Transect in the x-direction with 3 trees, VY = Variable Area Transect in the y-direction with 3 trees.

In figure 4, the results for the simulation-based study with tree densities of 0.2 trees/m^2^ are shown in a graph. In the random patterns, all methods score relatively well: The true density was included within the 95% confidence limits of all plotless methods tested.

In the aggregated patterns, all methods perform worse with no mean error smaller than 45%. The sign of the error in aggregated patterns is negative: the estimated density is much lower than the exact value. For random sampling points, the chance is higher to fall outside a cluster than within: the distance measured, which will also be squared, becomes larger when the sampling point falls between clusters and the estimated density will be lower than the exact density. For the Nearest Neighbour method the exact opposite mechanism operates: in an aggregated pattern the chance of a random tree falling within a cluster becomes larger while the distances within a cluster between trees are much smaller: the estimated density will be higher than the exact density.

In regular patterns, the Nearest Neighbour method gives an underestimation while the others overestimate the density. In regular patterns the variation in errors is mixed: the higher order methods (Ordered Distance 2, Ordered Distance 3, PCQM 2, PCQM 3 and VAT X and Y) perform relatively well with mean errors lower than 20%. On average, the gradient increases the error for all three patterns and methods selected.

#### Varying the tree density

The effect of varying the tree densities can be seen in chapter three of the supplementary material. The tables presented show that the error in the density estimate becomes (on average) smaller with increasing density. Only when a trend is present, the accuracy becomes less with higher densities, probably because the appearance of the trend also becomes stronger. Under clustered patterns, a lower density seems to result in a larger error, probably because the degree of aggregation also increases. Overall the Variable Area Transect Method (VAT) seems to perform best under varying tree density.

## Discussion

### *Observation-based Study*

In the field, mean underestimations and overestimations up to 97% and 53% respectively were found. These errors can result in estimated densities up to 30 times below the real value. Our findings confirm and extend results from other authors [12], [13], [28]. Errors as large as found here, based on actual field data, were however not yet reported. This is probably due to the species zonation present in the mangrove forest. One should remember though, that density gradients do occur outside mangroves. Often these gradients depend on changes in the environment like altitude. Therefore, these results apply to more types of vegetation.

### Simulation-based Study

For almost all methods the sign of the error - over or underestimation - depends on the pattern (aggregation or regular pattern). This mechanism will increase the error when tree densities of two forests with differing vegetation patterns are compared. The unreliability range then corresponds with the entire error range.

The patterns of our field sites vary between clustered and close to random. Sites 3 and 4 have patterns most closely approaching randomness. All methods give the highest accuracy for these sites (figure 3), just like for the random dispersion patterns in figure 4. The trees in site 1 and 2 show a more diverse pattern with also less accurate density estimations. Interestingly, the Nearest Neighbour method gives relatively good results in the field study, especially in site 1, while it gives worse results for the vegetation patterns. A possible explanation could be that both *Avicennia marina* and *Ceriops tagal* have a mix of vegetation patterns (both aggregation and regularity) in which the Nearest Neighbour method happens to perform well.

In the field, vegetation most probably displays a mix of patterns: a forest can display both aggregation and randomness at different sites, or even random aggregations or aggregations of random clusters.

#### Varying tree density

While varying the tree density under random and semi-regular patterns is rather straight forward, with aggregated patterns the question is what will happen with the size of the clusters: Will the density within tree clusters remain the same with varying tree density, or will it also vary? For our study we assumed that the density within the tree clusters remains similar. This causes a higher a lower R index; a higher degree of aggregation. Consequently the error in the density estimates for aggregated vegetation patterns is much higher. This could be an effect of the assumption made (similar within cluster densities), rather than an observation which could also be made in the field. These results therefore ask for more elaboration and research and concise conclusions upon the effect of density variations cannot be made for now.

#### Reflection

None of the plotless sampling methods gave good results across all study sites and vegetation patterns. Taking a combination of methods might be an alternative. White et al. [13] found a combined estimator taking the mean of two Nearest Neighbour methods and one Basic Distance method to perform the best. This combination however is very difficult to apply in the field. According to their study, the 2^nd^ and 3^rd^ order PCQM ranked highest for the clumped distribution and the Variable Area Transect method performed moderately well overall. Comparatively, our analysis does show better performance for higher order methods like PCQM 2 and 3 and theVariable Area Transect method. The difficulty lies with aggregation and zonation patterns, as these methods still give errors up to 80% in these circumstances. This gives very low certainty using these methods when vegetation patterns are unknown.

An alternative to field sampling is the use of remote sensing. Today interpretation still needs to be grounded in field data. Nagendra *et al.* [29] made an extensive review and stress the importance of in situ data for accurate interpretation of imagery. Studies in mangroves show that data from field surveys and remotely sensed data do not always correspond (for example due to overgrowth of one species by another) [20], [30] and that different data sources should be combined. With the advance of technological developments, one day remote sensing alone might be able to monitor forest resources. In the meantime, errors might be inevitable, but ignorance of uncertainty is avoidable.

This study has shown large errors in vegetation survey methods currently used in four field sites. These field sites are small and do not represent the large and diverse mangrove communities which can be found around the world. Using plotless sampling within different vegetation patterns shows that especially bad results are obtained within non-randomly dispersed vegetation. Therefore, it is important to find out what proportion of mangroves worldwide is characterised by a random spatial distribution of trees.

Caution should be taken when applying plotless sampling methods in the field particularly when used for societal purposes. Currently more than 90 percent of the world’s mangrove forests are located in developing countries [3]. Like elsewhere, the local community in our study site obtains direct benefits from the forest in terms of wood and non-wood products. Indirect benefits may come in the future when carbon sequestration by natural and replanted forests is valued and paid for. Using ground-based forest inventory approaches, the error generated in the tree density estimation multiplies with uncertainties in other variables. Inaccurate estimates, as can easily be generated by inappropriate methods, can make society pay too much or the community receive too little.

### Conclusions

#### Using plotless sampling in the field

The most important reason why plotless sampling techniques are being used is the relative ease compared with plot-based methods. This reflects the trade-off between maximum accuracy and minimum time required. Because plotless methods give the largest bias when vegetation has a high degree of non-randomness, we would like to recommend not using these methods when this is a known vegetation feature beforehand.

When the spatial pattern is unknown, it could be a rational approach to use a mix of two methods - for example plot-based and plotless - or two plotless methods with known contrasting biases - like Nearest Neighbour and Basic Distance. In all cases we recommend: i) To check results wherever possible with a second method; ii) To use a higher order of method (so Ordered Distance 2 instead of Ordered Distance 1 and PCQM 2 instead of PCQM 1) whenever possible; iii) To choose methods known to have a relative higher accuracy under varying conditions like the Variable Area Transect (VAT) method.

### Ideas for Further Research

This study has given some unexpected results and therefore there are several possibilities for further research. Preferably, we would like to find a sampling method that is easy and fast to use while giving reliable results. As this is not within reach at the moment, reconsidering plot-based methods, by making a large-scale comparison both on accuracy obtained and feasibility is a first recommendation.

Given the practical advantages in the field, a re-examination of the theoretical basis of plotless sampling might be helpful in reducing inherent calculation biases. Bouldin [31] makes a start with this and hopefully this can be extended. Hence our second recommendation is to make a deeper investigation into the relation between the combinations of spatial patterns and the accuracy for each method.

An additional factor might be that the tree density itself can also influence the accuracy of the sampling methods, as suggested by Engeman *et al.* [12] and White *et al.* [13], depending on spatial dispersion and estimator type. In our study, we have made a preliminary assessment of the possible effect of varying densities on the accuracy of our density estimations, as can be found in File S3. We hope that other researchers can extend these preliminary insights to a more detailed investigation.

Because the accuracy of plotless sampling depends on the randomness of the vegetation dispersion, there is a need for future assessments with spatial mapping of trees in mangrove forests. Our final recommendation is that more research can be executed which investigates the degree of randomness of mangrove trees in different environments and for different species around the world. Correlations with biophysical factors could allow for linkages with more fundamental research.

## Supporting Information

File S1 **MATLAB codes of the sampling methods.** For each plotless sampling method an algorithm was developed in MATLAB which could applied to any data matrix presenting either a forest or a vegetation pattern. In this datamatrix each tree should be represented by an x-coordinate and an y-coordinate with the x-coordinate in the first column and the y-coordinate in the second column of the same row.
(DOC)

File S2 **Mean density estimates with different number of sampling points.** To test the influence of the number of sampling points on the accuracy of each method tested, this number was varied between 5 and 30 random sampling points. Both the mean estimation and standard variation for the different values are are shown in 12 figures, one for each site and pattern.
(DOC)

File S3 **Effect of density itself on mean density estimations.** The vegetation patterns used in this study had a density of 0.2 trees/m^2^. To test the influence of the choice of this density on the results, we also varied the density between 0.05 and 1 trees/m^2^. The results found are presented in 6 tables, one for each vegetation pattern.
(DOC)
