# Supplementary material for: An Evaluation of Plotless Sampling Using Vegetation Simulations and Field Data from a Mangrove Forest
Source: PLoS One. 2013 Jun 27;8(6):e67201. doi: 10.1371/journal.pone.0067201 (PMC3695089; doi:10.1371/journal.pone.0067201)
Supplement: File S1 — MATLAB codes of the sampling methods. For each plotless sampling method an algorithm was developed in MATLAB which could applied to any data matrix presenting either a forest or a vegetation pattern. In this datamatrix each tree should be represented by an x-coordinate and an y-coordinate with the x-coordinate in the first column and the y-coordinate in the second column of the same row. (DOC) [file pone.0067201.s001.doc]

**MATLAB codes of the plotless sampling methods**

# Table of contents

[0. Table of contents 1](#__RefHeading___Toc357599043)

[1. Nearest Neighbour method (NN) 1](#__RefHeading___Toc357599044)

[2. Basic Distance method (BD) 3](#__RefHeading___Toc357599048)

[3. Ordered Distance method (OD) 4](#__RefHeading___Toc357599053)

[3.1 OD1 4](#__RefHeading___Toc357599059)

[3.2 OD2 5](#__RefHeading___Toc357599060)

[3.3 OD3 6](#__RefHeading___Toc357599068)

[4. Point Centered Quarter Method (PCQM) 8](#__RefHeading___Toc357599074)

[4.1 PCQM 1 8](#__RefHeading___Toc357599081)

[4.2 PCQM 2 11](#__RefHeading___Toc357599089)

[4.3 PCQM3 14](#__RefHeading___Toc357599098)

[5. Variable Area Transect (VAT) method 17](#__RefHeading___Toc357599105)

[5.1 VAT in the x-direction to the third tree at each sampling point (VAT X3) 17](#__RefHeading___Toc357599112)

[5.2 VAT in the y-direction to the third tree at each sampling point (VAT Y3) 19](#__RefHeading___Toc357599121)

[6. References cited 20](#__RefHeading___Toc357599122)

# Nearest Neighbour method (NN)

| **Method** | **distance(s) measured** | **Equation** | **Literature source** |
| --- | --- | --- | --- |
| Nearest neighbour | The distance between a tree and the nearest tree is measured. |  | , , |

function [Z] = nearestneighbour(M,lengthx,lengthy, number)

%function [Z] = nearestneighbour(M, lengthx,lengthy, number)

% nearestneighbour = Nearest Neighbour, density calculation following Cottam and Curtis 1956

% input : M = datamatrix, columns in datamatrix should be ordered as follows: 1 : x, 2 : y, 3 : stem area

% lengthx = length of x-axis plot in meters

% lengthy = length of y-axis plot in meters

% number = amount of random starting points

% output : Z Z(1) = density Z(2) is basal area

% density gives density found in number per squared meter

% basalarea gives stem area in squared meters per hectare

R = zeros(number,2); % matrix 2 columns of random points, first column x, second column y

P = zeros(size(M,4)); % matrix distances + number + x-point + y-point

T = zeros(number,2); % matrix 2 columns of random trees, first column x, second column y

N = zeros(size(M,1)); % matrix distances

A = zeros(number,1); % matrix shortest distances

B = zeros(number,1); % matrix stemareas closest individual

C = zeros(number,1); % matrix stemareas nearest neighbour

Z = zeros(1,2);

d = 0.05*sqrt(lengthx*lengthy); % distance from border plot at which no sampling points are taken

for a = 1: number % matrix R is a collection of random points in plot

R(a,1) = d + (lengthx-2*d)*rand(1); % x-coordinate of random point in plot

R(a,2) = d + (lengthy-2*d)*rand(1); % y-coordinate of random point in plot

end

for b = 1: number % This algorithm makes matrix T consisting of the trees closest to the random points - the closest individuals

for a = 1:size(M,1) % M = matrix plot

P(a,1) = sqrt(((R(b,1)-M(a,1))^2)+(R(b,2)-M(a,2))^2); % calculates distance from tree at row a to random point

P(a,2) = M(a,3); % stemarea

P(a,3) = M(a,1);

P(a,4) = M(a,2);

end

Q = sortrows(P,1); % matrix of sorted distances

T(b,1) = Q(1,3); % matrix T with 2 columns, first column x-coordinate, second column y-coordinate

T(b,2) = Q(1,4);

B(b) = Q(1,2); % stemarea

end

for b = 1: number % This algorithm calculates the distances from the trees in T to their nearest neighbours

for a = 1:size(M,1) % M = matrix plot

N(a,1) = sqrt(((T(b,1)-M(a,1))^2)+(T(b,2)-M(a,2))^2); % calculates distances from tree at row a in matrix M to tree in matrix T

N(a,2) = M(a,3); % stemarea

end

O = sortrows(N,1); % matrix of sorted distances

A(b) = O(2,1); % = shortest distance

C(b) = O(2,2); % = stemarea

end

sumdistances = sum(A);

density = 1/(2.778*(sumdistances/number)^2);

sumstemareas = sum(B)+sum(C);

averagestemarea = sumstemareas/(2*number);

basalarea = averagestemarea*density*10000;

Z(1,1) = density;

Z(1,2) = basalarea;

end

# Basic Distance method (BD)

| **Method** | **Description of distance(s) measured** | **Equation** | **Literature source** |
| --- | --- | --- | --- |
| Basic distance | The distance between a point and the nearest tree is measured. | $\hat{D}=\frac{1}{4*(\sum R_{(1)}/N)^{2}}$ |  |

function [Z] = BasicDistance(M,lengthx,lengthy,number)

% function [density] = BasicDistance(M,lengthx,lengthy,number)

% BasicDistance = Ordered Distance 1, density calculation following Cottam 1956

% input : M = datamatrix, columns in datamatrix should be ordered as follows: 1 : x, 2 : y, 3 : stem area

% lengthx = length of x-axis plot

% lengthy = length of y-axis plot

% number = amount of random starting points

% output : Z Z(1) = density, Z(2) = basalarea

% density gives density found in number per squared meter

% basalarea gives squared meter of stem area per hectare

R = zeros(number,2); % matrix 2 columns of random points, first column x, second column y

N = zeros(size(M,1),2); % matrix distances

A = zeros(number,1); % matrix shortest distances

B = zeros(number,1); % matrix stem areas

Z = zeros(1,2);

d = 0.05*sqrt(lengthx*lengthy); % distance from border plot at which no sampling points are taken

for a = 1: size(R,1) % matrix R is a collection of random points in plot

R(a,1) = d + (lengthx-2*d)*rand(1); % x-coordinate of random point in plot

R(a,2) = d + (lengthy-2*d)*rand(1); % y-coordinate of random point in plot

end

for b = 1: size(R,1)

for a = 1:size(M,1) % M = matrix plot

N(a,1) = sqrt(((R(b,1)-M(a,1))^2)+(R(b,2)-M(a,2))^2); % calculates distance from tree at row a to random point

N(a,2) = M(a,3);

end

O = sortrows(N,1); % matrix of sorted distances

A(b) = O(1,1);

B(b) = O(1,2);

end

sumdistances = sum(A);

density = 1/(4*(sumdistances/number)^2);

sumstemareas = sum(B);

averagestemarea = sumstemareas/number;

basalarea = averagestemarea*density*10000;

Z(1) = density;

Z(2) = basalarea;

end

# Ordered Distance method (OD)

| **Method** | **Description of distance(s) measured** | **Equation** | **Literature source** |
| --- | --- | --- | --- |
| Ordered distance | The distance between a point and the g^th^ nearest tree is measured. | $\hat{D}=\frac{g*n-1}{\pi\sum_{i} R^{2}}$ | , |

## 3.1 OD1

function [Z] = OrderedDistance1(M,lengthx,lengthy,number)

% function [density] = OrderedDistance1(M,lengthx,lengthy,number)

% OrderedDistance1 = Ordered Distance, density calculation following Steinke & Hennenberg 2006

% input : M is the datamatrix, columns in datamatrix should be ordered as follows: 1 : x, 2 : y, 3: stem area

% lengthx = length of x-axis plot

% lengthy = length of y-axis plot

% number = amount of random starting points

% output : Z Z(1) = density, Z(2) = basalarea

% density gives density found

% basalarea gives stem area in squared meters per hectare

R = zeros(number,2); % matrix 2 columns of random points, first column x, second column y

N = zeros(size(M,1),2); % matrix distances

A = zeros(number,1); % vector shortest distances

B = zeros(number,1); % vector stem areas

Q = zeros(number,1); % vector shortest distances squared

Z = zeros(1,2);

d = 0.05*sqrt(lengthx*lengthy); % distance from border plot at which no sampling points are taken

for a = 1: size(R,1) % matrix R is a collection of random points in plot

R(a,1) = d + (lengthx-2*d)*rand(1); % x-coordinate of random point in plot

R(a,2) = d + (lengthy-2*d)*rand(1); % y-coordinate of random point in plot

end

for b = 1: size(R,1)

for a = 1:size(M,1) % M = matrix plot

N(a,1) = sqrt(((R(b,1)-M(a,1))^2)+(R(b,2)-M(a,2))^2); % calculates distance from tree at row a to random point

N(a,2) = M(a,3);

end

O = sortrows(N); % matrix of sorted distances

A(b) = O(1,1);

B(b) = O(1,2);

end

for c = 1: size(A,1) % matrix shortest distances squared

Q(c) = (A(c))^2;

end

sumdistancessquared = sum(Q);

density = (number -1)/(pi*sumdistancessquared);

sumstemareas = sum(B);

averagestemarea = sumstemareas/number;

basalarea = averagestemarea*density*10000;

Z(1) = density;

Z(2) = basalarea;

End

## 3.2 OD2

function [Z] = OrderedDistance2(M,lengthx,lengthy,number)

% function [density] = OrderedDistance2Steinke(M,lengthx,lengthy,number)

% OrderedDistance2 = Ordered Distance 2nd tree, density calculation following Steinke & Hennenberg 2006

% input : M is the datamatrix, columns in datamatrix should be ordered as follows: 1 : x, 2 : y, 3: stem area

% lengthx = length of x-axis plot

% lengthy = length of y-axis plot

% number = amount of random starting points

% output : Z Z(1) = density, Z(2) = basalarea

% density gives density found

% basalarea gives stem area in squared meters per hectare

R = zeros(number,2); % matrix 2 columns of random points, first column x, second column y

N = zeros(size(M,1),2); % matrix distances

A = zeros(number,1); % matrix shortest distances

B = zeros(number,1); % vector stem areas

Q = zeros(number,1); % matrix shortest distances squared

Z = zeros(1,2);

d = 2*(0.05*sqrt(lengthx*lengthy)); % distance from border plot at which no sampling points are taken

for a = 1: size(R,1) % matrix R is a collection of random points in plot

R(a,1) = d + (lengthx-2*d)*rand(1); % x-coordinate of random point in plot

R(a,2) = d + (lengthy-2*d)*rand(1); % y-coordinate of random point in plot

end

for b = 1: size(R,1)

for a = 1:size(M,1) % M = matrix plot

N(a,1) = sqrt(((R(b,1)-M(a,1))^2)+(R(b,2)-M(a,2))^2); % calculates distance from tree at row a to random point

N(a,2) = M(a,3);

end

O = sortrows(N,1); % matrix of sorted distances

A(b) = O(2,1); % second shortest distance is stored in matrix A

B(b) = O(2,2); % stem area

end

for c = 1: size(A,1) % This algorithm makes a vector Q with the distances squared

Q(c) = (A(c))^2;

end

sumdistancessquared = sum(Q);

density = (2*number -1)/(pi*sumdistancessquared);

sumstemareas = sum(B);

averagestemarea = sumstemareas/number;

basalarea = averagestemarea*density*10000;

Z(1) = density;

Z(2) = basalarea;

end

## 3.3 OD3

function [Z] = OrderedDistance3(M,lengthx,lengthy,number)

% function [density, basalarea] = OrderedDistance3(M,lengthx,lengthy,number)

% OrderedDistance3 = Ordered Distance 3rd tree, density calculation following Steinke & Hennenberg 2006

% input : M is the datamatrix, columns in datamatrix should be ordered as follows: 1 : x, 2 : y, 3: stem area

% lengthx = length of x-axis plot

% lengthy = length of y-axis plot

% number = amount of random starting points

% output : Z Z(1) = density, Z(2) = basalarea

% density gives density found

% basalarea gives stem area in squared meters per hectare

R = zeros(number,2); % matrix 2 columns of random points, first column x, second column y

N = zeros(size(M,1),2); % matrix distances

A = zeros(number,1); % vector shortest distances

B = zeros(number,1); % vector stem areas

Q = zeros(number,1); % vector shortest distances squared

Z = zeros(1,2);

d = 3*(0.05*sqrt(lengthx*lengthy)); % distance from border plot at which no sampling points are taken

for a = 1: size(R,1) % matrix R is a collection of random points in plot

R(a,1) = d + (lengthx-2*d)*rand(1); % x-coordinate of random point in plot

R(a,2) = d + (lengthy-2*d)*rand(1); % y-coordinate of random point in plot

end

for b = 1: size(R,1)

for a = 1:size(M,1) % M = matrix plot

N(a,1) = sqrt(((R(b,1)-M(a,1))^2)+(R(b,2)-M(a,2))^2); % calculates distance from tree at row a to random point

N(a,2) = M(a,3);

end

O = sortrows(N,1); % matrix of sorted distances

A(b) = O(3,1); % third shortest distance is stored in matrix A

B(b) = O(3,2);

end

for c = 1: size(A,1) % This algorithm makes a vector Q with the distances squared

Q(c) = (A(c))^2;

end

sumdistancessquared = sum(Q);

density = (3*number -1)/(pi*sumdistancessquared);

sumstemareas = sum(B);

averagestemarea = sumstemareas/number;

basalarea = averagestemarea*density*10000;

Z(1) = density;

Z(2) = basalarea;

end

# Point Centered Quarter Method (PCQM)

| **Method** | **Description of distance(s) measured** | **Equation** | **Literature source** |
| --- | --- | --- | --- |
| PCQM | The distances between a point and the g^th^ nearest tree in each quadrant around the point is measured. | $\hat{D}=\frac{k(gkn-1)}{\pi\sum_{i=1}^{n} \sum_{j=1}^{4} R_{ij}^{2}}$ | , |

## 4.1 PCQM 1

function [Z] = PCQM_mono(M,lengthx,lengthy,number)

% function [density] = PCQM_mono(M,lengthx,lengthy,number)

% PCQM following Mitchell 2007, first observation of particular species in each quadrant.

% input : M is the datamatrix, columns in datamatrix should be ordered as follows: 1 : x, 2 : y, 3 : stem area, 4 : species

% lengthx = length of x-axis plot in meters

% lengthy = length of y-axis plot in meters

% number = amount of random starting points

% output : Z Z(1) = density, Z(2) = basalarea, Z(3) = newnumber

% density gives density found in number per squared meter

% basalarea gives squared meter of stem area per hectare

% newnumber gives the amount of random points that are used for the calculation

savenumber = number*5;

R = zeros(savenumber,2); % matrix with 2 columns of random points, first column x, second column y

N = zeros(size(M,1),2);% matrix with distances

LB = zeros(size(M,1),2); % LB = upper left quadrant

RB = zeros(size(M,1),2); % RB = upper right quadrant

LO = zeros(size(M,1),2); % LO = lower left quadrant

RO = zeros(size(M,1),2); % RO = lower right quadrant

LBO = zeros(1,2);

RBO = zeros(1,2);

LOO = zeros(1,2);

ROO = zeros(1,2);

A = zeros(savenumber,1);

B = zeros(savenumber,1);

C = zeros(savenumber,1);

D = zeros(savenumber,1);

E = zeros(savenumber,1);

F = zeros(savenumber,1);

G = zeros(savenumber,1);

H = zeros(savenumber,1);

Z = zeros(1,2);

for a = 1: savenumber % This algorithm makes matrix R with a collection of random points in plot

R(a,1) = lengthx*rand(1); % x-coordinate of random point in plot

R(a,2) = lengthy*rand(1); % y-coordinate of random point in plot

end

g=1;

h=1;

k=1;

m=1;

for b = 1: savenumber

for a = 1:size(M,1) % M = matrix plot

N(a,1) = sqrt(((R(b,1)-M(a,1))^2)+(R(b,2)-M(a,2))^2); % calculates distance from tree at row a to random point

if (M(a,1) < R(b,1)) && (M(a,2) >= R(b,2)) % denotes all trees in upper left quadrant to LB

LB(a,1) = N(a,1);

LB(a,2) = M(a,3); % basal area

end

if (M(a,1) >= R(b,1)) && (M(a,2) > R(b,2)) % denotes all trees in upper right quadrant to RB

RB(a,1) = N(a,1);

RB(a,2) = M(a,3); % basal area

end

if (M(a,1) <= R(b,1)) && (M(a,2) < R(b,2)) % denotes all trees in lower left quadrant to LO

LO(a,1) = N(a,1);

LO(a,2) = M(a,3);

end

if (M(a,1) > R(b,1)) && (M(a,2) <= R(b,2)) % denotes all trees in lower right quadrant to RO

RO(a,1) = N(a,1);

RO(a,2) = M(a,3);

end

end

for f = 1: size(LB,1) % makes matrix LBO without values zero

if LB(f,1)>0

LBO(g,1) = LB(f,1);

LBO(g,2) = LB(f,2);

g = g+1;

end

end

for f = 1: size(RB,1) % makes matrix RBO without values zero

if RB(f,1)>0

RBO(h,1) = RB(f,1);

RBO(h,2) = RB(f,2);

h = h+1;

end

end

for f = 1: size(LO,1) % makes matrix LOO without values zero

if LO(f,1)>0

LOO(k,1) = LO(f,1);

LOO(k,2) = LO(f,2);

k = k+1;

end

end

for f = 1: size(RO,1) % makes matrix ROO without values zero

if RO(f,1)>0

ROO(m,1) = RO(f,1);

ROO(m,2) = RO(f,2);

m = m+1;

end

end

LBsort = sortrows(LBO,1); % sorts matrices according to the first column - the distances

RBsort = sortrows(RBO,1);

LOsort = sortrows(LOO,1);

ROsort = sortrows(ROO,1);

if ((LBsort(1,1)) > 0) && ((RBsort(1,1)) > 0) && ((LOsort(1,1)) > 0) && ((ROsort(1,1)) > 0) % On the condition that there is a tree in each quadrant

A(b) = LBsort(1,1); % assigns the distances to 4 vectors

B(b) = RBsort(1,1);

C(b) = LOsort(1,1);

D(b) = ROsort(1,1);

E(b) = LBsort(1,2); % assigns the stem areas to 4 vectors

F(b) = RBsort(1,2);

G(b) = LOsort(1,2);

H(b) = ROsort(1,2);

%K(b)= 1;

end

LB = zeros(size(M,1),2); % empties the quadrant matrices for new use with new random point

RB = zeros(size(M,1),2);

LO = zeros(size(M,1),2);

RO = zeros(size(M,1),2);

LBO = zeros(1,2);

RBO = zeros(1,2);

LOO = zeros(1,2);

ROO = zeros(1,2);

g = 1;

h = 1;

k = 1;

m = 1;

end

P = (A>0);

Anew = A(P);

Bnew = B(P);

Cnew = C(P);

Dnew = D(P);

Enew = E(P);

Fnew = F(P);

Gnew = G(P);

Hnew = H(P);

Anumber = Anew(1:number,1);

Bnumber = Bnew(1:number,1);

Cnumber = Cnew(1:number,1);

Dnumber = Dnew(1:number,1);

Enumber = Enew(1:number,1);

Fnumber = Fnew(1:number,1);

Gnumber = Gnew(1:number,1);

Hnumber = Hnew(1:number,1);

AA = Anumber.^2; % to the power 2 (zeros end up as zeros anyway)

BB = Bnumber.^2;

CC = Cnumber.^2;

DD = Dnumber.^2;

sumall = sum(AA) + sum(BB) + sum(CC) + sum(DD);

density = (4*(4*number - 1))/(pi()*sumall);

sumstemareas = sum(Enumber) + sum(Fnumber) + sum(Gnumber) + sum(Hnumber);

averagestemarea = sumstemareas/(4*number);

basalarea = averagestemarea*density*10000; % calculates basal area

Z(1) = density;

Z(2) = basalarea;

end

## 4.2 PCQM 2

function [density] = PCQM2Pollard(M,lengthx,lengthy,number)

% function [density] = PCQM_mono(M,lengthx,lengthy,number)

% PCQM following Pollard 1971, second observation of particular species in each quadrant.

% input : M is the datamatrix, columns in datamatrix should be ordered as follows: 1 : x, 2 : y, 3 : stem area, 4 : species

% lengthx = length of x-axis plot in meters

% lengthy = length of y-axis plot in meters

% number = amount of random starting points

% output : density found in number per squared meter

savenumber = number*5;

R = zeros(savenumber,2); % matrix with 2 columns of random points, first column x, second column y

N = zeros(size(M,1),2);% matrix with distances

LB = zeros(size(M,1),2); % LB = upper left quadrant

RB = zeros(size(M,1),2); % RB = upper right quadrant

LO = zeros(size(M,1),2); % LO = lower left quadrant

RO = zeros(size(M,1),2); % RO = lower right quadrant

LBO = zeros(2,2);

RBO = zeros(2,2);

LOO = zeros(2,2);

ROO = zeros(2,2);

A = zeros(savenumber,1);

B = zeros(savenumber,1);

C = zeros(savenumber,1);

D = zeros(savenumber,1);

%E = zeros(savenumber,1);

%F = zeros(savenumber,1);

%G = zeros(savenumber,1);

%H = zeros(savenumber,1);

%Z = zeros(1,2);

for a = 1: savenumber % This algorithm makes matrix R with a collection of random points in plot

R(a,1) = lengthx*rand(1); % x-coordinate of random point in plot

R(a,2) = lengthy*rand(1); % y-coordinate of random point in plot

end

g=1;

h=1;

k=1;

m=1;

for b = 1: savenumber

for a = 1:size(M,1) % M = matrix plot

N(a,1) = sqrt(((R(b,1)-M(a,1))^2)+(R(b,2)-M(a,2))^2); % calculates distance from tree at row a to random point

if (M(a,1) < R(b,1)) && (M(a,2) >= R(b,2)) % denotes all trees in upper left quadrant to LB

LB(a,1) = N(a,1);

%LB(a,2) = M(a,3); % basal area

end

if (M(a,1) >= R(b,1)) && (M(a,2) > R(b,2)) % denotes all trees in upper right quadrant to RB

RB(a,1) = N(a,1);

%RB(a,2) = M(a,3); % basal area

end

if (M(a,1) <= R(b,1)) && (M(a,2) < R(b,2)) % denotes all trees in lower left quadrant to LO

LO(a,1) = N(a,1);

%LO(a,2) = M(a,3);

end

if (M(a,1) > R(b,1)) && (M(a,2) <= R(b,2)) % denotes all trees in lower right quadrant to RO

RO(a,1) = N(a,1);

%RO(a,2) = M(a,3);

end

end

for f = 1: size(LB,1) % makes matrix LBO without values zero

if LB(f,1)>0

LBO(g,1) = LB(f,1);

%LBO(g,2) = LB(f,2);

g = g+1;

end

end

for f = 1: size(RB,1) % makes matrix RBO without values zero

if RB(f,1)>0

RBO(h,1) = RB(f,1);

%RBO(h,2) = RB(f,2);

h = h+1;

end

end

for f = 1: size(LO,1) % makes matrix LOO without values zero

if LO(f,1)>0

LOO(k,1) = LO(f,1);

%LOO(k,2) = LO(f,2);

k = k+1;

end

end

for f = 1: size(RO,1) % makes matrix ROO without values zero

if RO(f,1)>0

ROO(m,1) = RO(f,1);

%ROO(m,2) = RO(f,2);

m = m+1;

end

end

LBsort = sortrows(LBO,1); % sorts matrices according to the first column - the distances

RBsort = sortrows(RBO,1);

LOsort = sortrows(LOO,1);

ROsort = sortrows(ROO,1);

if ((LBsort(2,1)) > 0) && ((RBsort(2,1)) > 0) && ((LOsort(2,1)) > 0) && ((ROsort(2,1)) > 0) % On the condition that there is a tree in each quadrant

A(b) = LBsort(2,1); % assigns the second distances to 4 vectors

B(b) = RBsort(2,1);

C(b) = LOsort(2,1);

D(b) = ROsort(2,1);

%E(b) = LBsort(1,2); % assigns the stem areas to 4 vectors

%F(b) = RBsort(1,2);

%G(b) = LOsort(1,2);

%H(b) = ROsort(1,2);

%K(b)= 1;

end

LB = zeros(size(M,1),2); % empties the quadrant matrices for new use with new random point

RB = zeros(size(M,1),2);

LO = zeros(size(M,1),2);

RO = zeros(size(M,1),2);

LBO = zeros(2,2);

RBO = zeros(2,2);

LOO = zeros(2,2);

ROO = zeros(2,2);

g = 1;

h = 1;

k = 1;

m = 1;

end

P = (A>0);

Anew = A(P);

Bnew = B(P);

Cnew = C(P);

Dnew = D(P);

%Enew = E(P);

%Fnew = F(P);

%Gnew = G(P);

%Hnew = H(P);

Anumber = Anew(1:number,1);

Bnumber = Bnew(1:number,1);

Cnumber = Cnew(1:number,1);

Dnumber = Dnew(1:number,1);

%Enumber = Enew(1:number,1);

%Fnumber = Fnew(1:number,1);

%Gnumber = Gnew(1:number,1);

%Hnumber = Hnew(1:number,1);

AA = Anumber.^2; % to the power 2 (zeros end up as zeros anyway)

BB = Bnumber.^2;

CC = Cnumber.^2;

DD = Dnumber.^2;

sumall = sum(AA) + sum(BB) + sum(CC) + sum(DD);

density = (4*(8*number-1))/(pi()*sumall);

%sumstemareas = sum(Enumber) + sum(Fnumber) + sum(Gnumber) + sum(Hnumber);

%averagestemarea = sumstemareas/(4*number);

%basalarea = averagestemarea*density*10000; % calculates basal area

end

## 4.3 PCQM3

function [density] = PCQM3Pollard(M,lengthx,lengthy,number)

% function [density] = PCQM_mono(M,lengthx,lengthy,number)

% PCQM following Pollard 1971, third observation of particular species in each quadrant.

% input : M is the datamatrix, columns in datamatrix should be ordered as follows: 1 : x, 2 : y, 3 : stem area, 4 : species

% lengthx = length of x-axis plot in meters

% lengthy = length of y-axis plot in meters

% number = amount of random starting points

% output : density found in number per squared meter

savenumber = number*5;

R = zeros(savenumber,2); % matrix with 2 columns of random points, first column x, second column y

N = zeros(size(M,1),2);% matrix with distances

LB = zeros(size(M,1),2); % LB = upper left quadrant

RB = zeros(size(M,1),2); % RB = upper right quadrant

LO = zeros(size(M,1),2); % LO = lower left quadrant

RO = zeros(size(M,1),2); % RO = lower right quadrant

LBO = zeros(3,2);

RBO = zeros(3,2);

LOO = zeros(3,2);

ROO = zeros(3,2);

A = zeros(savenumber,1);

B = zeros(savenumber,1);

C = zeros(savenumber,1);

D = zeros(savenumber,1);

%E = zeros(savenumber,1);

%F = zeros(savenumber,1);

%G = zeros(savenumber,1);

%H = zeros(savenumber,1);

%Z = zeros(1,2);

for a = 1: savenumber % This algorithm makes matrix R with a collection of random points in plot

R(a,1) = lengthx*rand(1); % x-coordinate of random point in plot

R(a,2) = lengthy*rand(1); % y-coordinate of random point in plot

end

g=1;

h=1;

k=1;

m=1;

for b = 1: savenumber

for a = 1:size(M,1) % M = matrix plot

N(a,1) = sqrt(((R(b,1)-M(a,1))^2)+(R(b,2)-M(a,2))^2); % calculates distance from tree at row a to random point

if (M(a,1) < R(b,1)) && (M(a,2) >= R(b,2)) % denotes all trees in upper left quadrant to LB

LB(a,1) = N(a,1);

%LB(a,2) = M(a,3); % basal area

end

if (M(a,1) >= R(b,1)) && (M(a,2) > R(b,2)) % denotes all trees in upper right quadrant to RB

RB(a,1) = N(a,1);

%RB(a,2) = M(a,3); % basal area

end

if (M(a,1) <= R(b,1)) && (M(a,2) < R(b,2)) % denotes all trees in lower left quadrant to LO

LO(a,1) = N(a,1);

%LO(a,2) = M(a,3);

end

if (M(a,1) > R(b,1)) && (M(a,2) <= R(b,2)) % denotes all trees in lower right quadrant to RO

RO(a,1) = N(a,1);

%RO(a,2) = M(a,3);

end

end

for f = 1: size(LB,1) % makes matrix LBO without values zero

if LB(f,1)>0

LBO(g,1) = LB(f,1);

%LBO(g,2) = LB(f,2);

g = g+1;

end

end

for f = 1: size(RB,1) % makes matrix RBO without values zero

if RB(f,1)>0

RBO(h,1) = RB(f,1);

%RBO(h,2) = RB(f,2);

h = h+1;

end

end

for f = 1: size(LO,1) % makes matrix LOO without values zero

if LO(f,1)>0

LOO(k,1) = LO(f,1);

%LOO(k,2) = LO(f,2);

k = k+1;

end

end

for f = 1: size(RO,1) % makes matrix ROO without values zero

if RO(f,1)>0

ROO(m,1) = RO(f,1);

%ROO(m,2) = RO(f,2);

m = m+1;

end

end

LBsort = sortrows(LBO,1); % sorts matrices according to the first column - the distances

RBsort = sortrows(RBO,1);

LOsort = sortrows(LOO,1);

ROsort = sortrows(ROO,1);

if ((LBsort(3,1)) > 0) && ((RBsort(3,1)) > 0) && ((LOsort(3,1)) > 0) && ((ROsort(3,1)) > 0) % On the condition that there is a tree in each quadrant

A(b) = LBsort(3,1); % assigns the second distances to 4 vectors

B(b) = RBsort(3,1);

C(b) = LOsort(3,1);

D(b) = ROsort(3,1);

%E(b) = LBsort(1,2); % assigns the stem areas to 4 vectors

%F(b) = RBsort(1,2);

%G(b) = LOsort(1,2);

%H(b) = ROsort(1,2);

%K(b)= 1;

end

LB = zeros(size(M,1),2); % empties the quadrant matrices for new use with new random point

RB = zeros(size(M,1),2);

LO = zeros(size(M,1),2);

RO = zeros(size(M,1),2);

LBO = zeros(3,2);

RBO = zeros(3,2);

LOO = zeros(3,2);

ROO = zeros(3,2);

g = 1;

h = 1;

k = 1;

m = 1;

end

P = (A>0);

Anew = A(P);

Bnew = B(P);

Cnew = C(P);

Dnew = D(P);

%Enew = E(P);

%Fnew = F(P);

%Gnew = G(P);

%Hnew = H(P);

Anumber = Anew(1:number,1);

Bnumber = Bnew(1:number,1);

Cnumber = Cnew(1:number,1);

Dnumber = Dnew(1:number,1);

%Enumber = Enew(1:number,1);

%Fnumber = Fnew(1:number,1);

%Gnumber = Gnew(1:number,1);

%Hnumber = Hnew(1:number,1);

AA = Anumber.^2; % to the power 2 (zeros end up as zeros anyway)

BB = Bnumber.^2;

CC = Cnumber.^2;

DD = Dnumber.^2;

sumall = sum(AA) + sum(BB) + sum(CC) + sum(DD);

density = (4*(12*number-1))/(pi()*sumall);

%sumstemareas = sum(Enumber) + sum(Fnumber) + sum(Gnumber) + sum(Hnumber);

%averagestemarea = sumstemareas/(4*number);

%basalarea = averagestemarea*density*10000; % calculates basal area

end

# Variable Area Transect (VAT) method

| **Method** | **Description of distance(s) measured** | **Equation** | **Literature source** |
| --- | --- | --- | --- |
| Variable Area Transect | The distance from a point to the g^th^ individual in a given direction with a certain width (a transect) is measured. | $\hat{D}=\frac{gn-1}{w\sum_{i} L_{(g)i}}$ |  |

## 5.1 VAT in the x-direction to the third tree at each sampling point (VAT X3)

function [Z] = VATX3(M, lengthx, lengthy, number)

% function [density] = VATX2(M,lengthx,lengthy,numberpoints, width, numbertrees)

% VAT following Parker 1979, all transects in the x-direction and until 3rd

% tree

% input : M is the datamatrix, columns in datamatrix should be ordered as follows: 1 : x, 2 : y, 3 : stem area

% lengthx = length of x-axis plot

% lengthy = length of y-axis plot

% numberpoints = amount of random starting points

% output : Z Z(1) = density, Z(2) = basalarea

% density gives density found

% basalarea gives stem area in squared meters per hectare

savenumber = number*3;

R = zeros(savenumber,2); % matrix 2 columns of random points, first column x, second column y

A = zeros(savenumber,1);

C = zeros(savenumber,1);

D = zeros(savenumber,1);

E = zeros(savenumber,1);

N = zeros(size(M,1),3); % matrix with in first column absolute difference in y-direction, second column x-point of tree and in third column stemarea of tree

O = zeros(3,2);

Z = zeros(2,1);

width = 4;

halfwidth = width/2;

for a = 1: savenumber % matrix R is a collection of random points in plot

R(a,1) = lengthx*rand(1); % x-coordinate of random point in plot

R(a,2) = halfwidth + (lengthy-width)*rand(1); % y-coordinate of random point in plot

end

g = 1;

for b = 1: savenumber

for a = 1:size(M,1) % matrix plot

if (M(a,1) > R(b,1)) && ((abs(M(a,2) - R(b,2))) < halfwidth) % if x-value of tree in plot is greater than x-value random point and the y-difference is smaller than half of the width of the transect

N(a,1) = M(a,1)-R(b,1);

N(a,2) = M(a,3);

end

end

for f = 1: size(N,1) % makes matrix O without values zero

if N(f,1)>0

O(g,1) = N(f,1);

O(g,2) = N(f,2);

g = g+1;

end

end

P = sortrows(O,1);

if P(3,1) > 0

A(b) = P(3,1); % third tree

%F(b) = 1;

C(b) = P(1,2);

D(b) = P(2,2);

E(b) = P(3,2);

end

N = zeros(size(M,1),2);

O = zeros(3,2);

g = 1;

end

R = (A>0);

Anew = A(R);

Anumber = Anew(1:number,1);

Cnew = C(R);

Dnew = D(R);

Enew = E(R);

Cnumber = Cnew(1:number,1);

Dnumber = Dnew(1:number,1);

Enumber = Enew(1:number,1);

sumlengths = sum(Anumber);

density = (3*number-1)/(width*sumlengths);

sumstemareas = sum(Cnumber) + sum(Dnumber) + sum(Enumber);

averagestemarea = sumstemareas/(3*number);

basalarea = averagestemarea*density*10000; % calculates basal area

Z(1) = density;

Z(2) = basalarea;

End

## 5.2 VAT in the y-direction to the third tree at each sampling point (VAT Y3)

function [Z] = VATY3(M, lengthx, lengthy, number)

% function [density] = VATX2(M,lengthx,lengthy,numberpoints, width, numbertrees)

% VAT following Parker 1979, all transects in the x-direction

% input : M is the datamatrix, columns in datamatrix should be ordered as follows: 1 : x, 2 : y, 3: height, 4 : stem area, 5: species

% lengthx = length of x-axis plot

% lengthy = length of y-axis plot

% numberpoints = amount of random starting points

% numbertrees = number of trees per transect

% output : Z(1) = density, Z(2) = basalarea, Z(3) = newnumber

% density gives density found

% basalarea gives stem area in squared meters per hectare

savenumber = number*3;

R = zeros(savenumber,2); % matrix 2 columns of random points, first column x, second column y

A = zeros(savenumber,1);

C = zeros(savenumber,1);

D = zeros(savenumber,1);

E = zeros(savenumber,1);

%F = zeros(numberpoints,1);

N = zeros(size(M,1),2);

O = zeros(3,2);

Z = zeros(2,1);

width = 4;

halfwidth = width/2;

for a = 1: savenumber % matrix R is a collection of random points in plot

R(a,1) = halfwidth + (lengthx-width)*rand(1); % x-coordinate of random point in plot

R(a,2) = lengthy*rand(1); % y-coordinate of random point in plot

end

g = 1;

for b = 1: savenumber

for a = 1:size(M,1) % matrix plot

if (M(a,2) > R(b,2)) && ((abs(M(a,1) - R(b,1))) < halfwidth) % if y-value of tree in plot is greater than y-value random point and the x-difference is smaller than half of the width of the transect

N(a,1) = M(a,2)-R(b,2); % difference in y-direction

N(a,2) = M(a,3);

end

end

for f = 1: size(N,1) % makes matrix O without values zero

if N(f,1)>0

O(g,1) = N(f,1); % O = matrix with only difference y-direction

O(g,2) = N(f,2);

g = g+1;

end

end

P = sortrows(O,1);

if P(3,1) > 0

A(b) = P(3,1); % y difference second tree

%F(b) = 1; % counts valid points

C(b) = P(1,2); % stem area first tree

D(b) = P(2,2); % stem area second tree

E(b) = P(3,2); % stem area third tree

end

N = zeros(size(M,1),2);

O = zeros(3,2);

g = 1;

end

R = (A>0);

Anew = A(R);

Anumber = Anew(1:number,1);

Cnew = C(R);

Dnew = D(R);

Enew = E(R);

Cnumber = Cnew(1:number,1);

Dnumber = Dnew(1:number,1);

Enumber = Enew(1:number,1);

sumlengths = sum(Anumber);

density = (3*number-1)/(width*sumlengths);

sumstemareas = sum(Cnumber) + sum(Dnumber) + sum(Enumber);

averagestemarea = sumstemareas/(3*number);

basalarea = averagestemarea*density*10000; % calculates basal area

Z(1) = density;

Z(2) = basalarea;

end

# References cited

1. Cottam G, Curtis JT (1956) The Use of Distance Measures in Phytosociological Sampling. Ecology 37: 451-460.

2. Engeman RM, Sugihara RT, Pank LF, Dusenberry WE (1994) A Comparison of Plotless Density Estimators Using Monte Carlo Simulation. Ecology 75: 1769-1779.

3. White N, Engeman R, Sugihara R, Krupa H (2008) A comparison of plotless density estimators using Monte Carlo simulation on totally enumerated field data sets. BMC Ecology 8: 6.

4. Pollard JH (1971) On Distance Estimators of Density in Randomly Distributed Forests. Biometrics 27: 991-1002.

5. Steinke I, Hennenberg KJ (2006) On the power of plotless density estimators for statistical comparisons of plant populations. Canadian Journal of Botany 84: 421-432.

6. Mitchell K (2007) Quantitative Analysis by the Point-Centered Quarter Method. Geneva: Department of Mathematics and Computer Science, Hobart and William Smith Colleges.

7. Parker KR (1979) Density Estimation by Variable Area Transect. The Journal of Wildlife Management 43: 484-492.
