# Supplementary material for: An Evaluation of Plotless Sampling Using Vegetation Simulations and Field Data from a Mangrove Forest
Source: PLoS One. 2013 Jun 27;8(6):e67201. doi: 10.1371/journal.pone.0067201 (PMC3695089; doi:10.1371/journal.pone.0067201)
Supplement: File S2 — Mean density estimates with different number of sampling points. To test the influence of the number of sampling points on the accuracy of each method tested, this number was varied between 5 and 30 random sampling points. Both the mean estimation and standard variation for the different values are are shown in 12 figures, one for each site and pattern. (DOC) [file pone.0067201.s002.doc]

**Mean density estimates with different number of sampling points**

# Table of contents

[0. Table of contents 1](#__RefHeading___Toc357600314)

[1. Introduction 1](#__RefHeading___Toc357600315)

[2. Sites 1](#__RefHeading___Toc357600316)

[3. Vegetation patterns 4](#__RefHeading___Toc357600317)

[4. References cited 6](#__RefHeading___Toc357600318)

# Introduction

In the literature , a minimum of 20 sampling points is recommended for plotless sampling methods. To check this minimum standard, we used for a variation between 5 and 30 sampling points for each method. Our analysis shows that a minimum of 15 sampling points gives a robust estimation.

This supporting online information gives twelve figures: For each tree species and site in the mangrove forest (6 combinations) the means of the density estimations and the standard deviations with 5 to 30 sampling points. For each vegetation dispersion pattern (6 patterns) the means of the density estimations and the standard deviations are also given for 5 to 30 sampling points.

As can be seen from these graphs, the results do not change much when more than 15 sampling points are used. To be sure that the number of sampling points does not influence the accuracy evaluated, in our study results we always give the results obtained with 30 sampling points.

# Sites

## 2.1 Estimates of Avicennia marina tree density in site 1


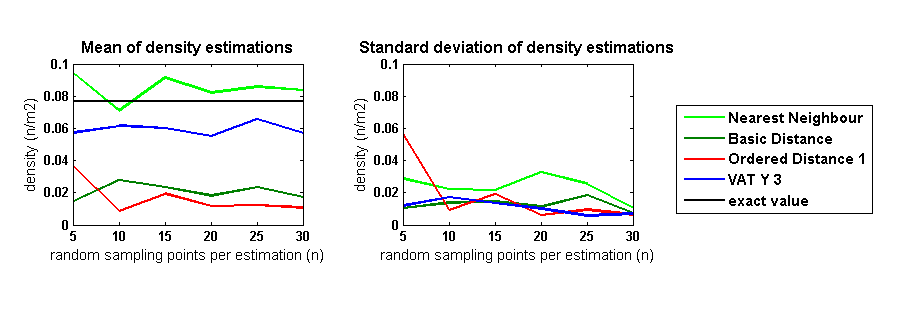


*Fig. 1)Means and standard deviations of tree density for* Avicennia marina *in site1* *are plotted against the number of random sampling points using plotless sampling techniques. A repetition factor of 10 was used for the calculation.*

## Estimates of Ceriops tagal tree density in site 1


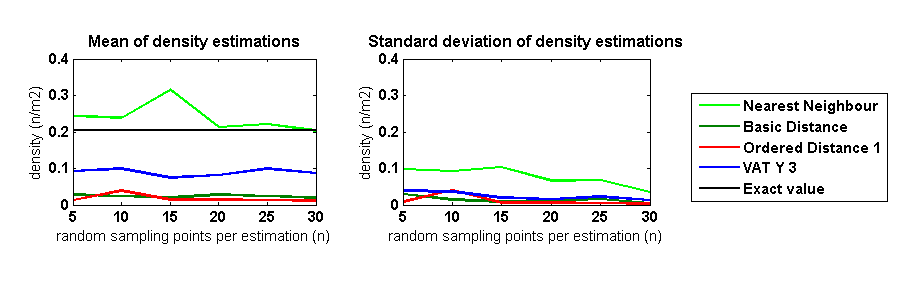


*Fig. 2)Means and standard deviations of tree density for* Ceriops tagal *in site 1* *are plotted against the number of random sampling points using plotless sampling techniques. A repetition factor of 10 was used for the calculation.*

## 2.3 Estimates of Ceriops tagal tree density in site 2


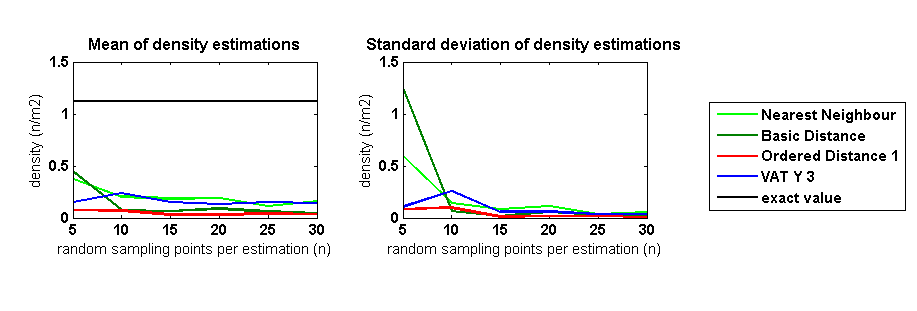


*Fig. 3)Means and standard deviations of tree density for* Ceriops tagal *in site 2* *are plotted against the number of random sampling points using plotless sampling techniques. A repetition factor of 10 was used for the calculation.*

## 2.4 Estimates of Rhizophora mucronata tree density in site 2


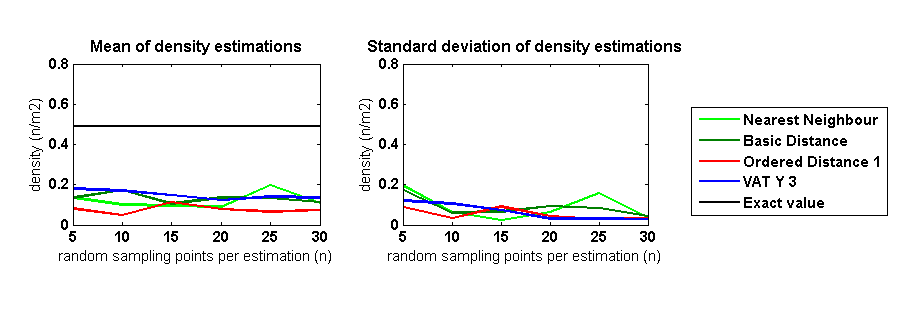


*Fig. 4)Means and standard deviations of tree density for* Rhizophora mucronata *in site 2* *are plotted against the number of random sampling points using plotless sampling techniques. A repetition factor of 10 was used for the calculation.*

## 2.5 Estimates of Avicennia marina tree density in site 3


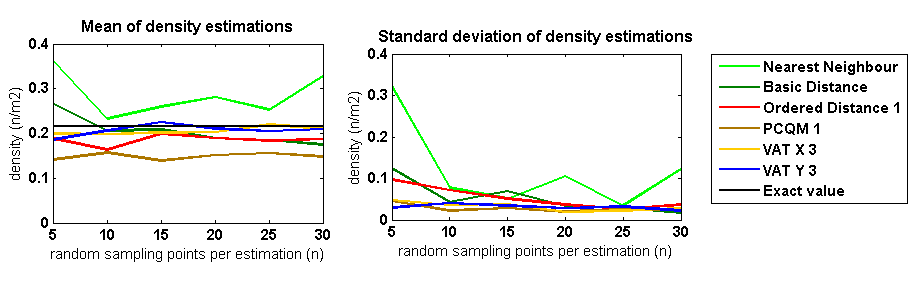


*Fig. 5)Means and standard deviations of tree density for* Avicennia marina *in site 3* *are plotted against the number of random sampling points using plotless sampling techniques. A repetition factor of 10 was used for the calculation.*

## 2.6 Estimates of Avicennia marina tree density in site 4


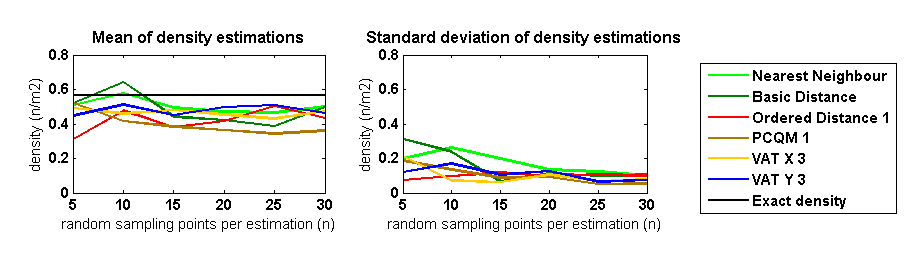


*Fig. 6)Means and standard deviations of tree density for* Avicennia marina *in site 4* *are plotted against the number of random sampling points using plotless sampling techniques. A repetition factor of 10 was used for the calculation.*

# Vegetation patterns

## 3.1 Estimates of tree density in a random pattern


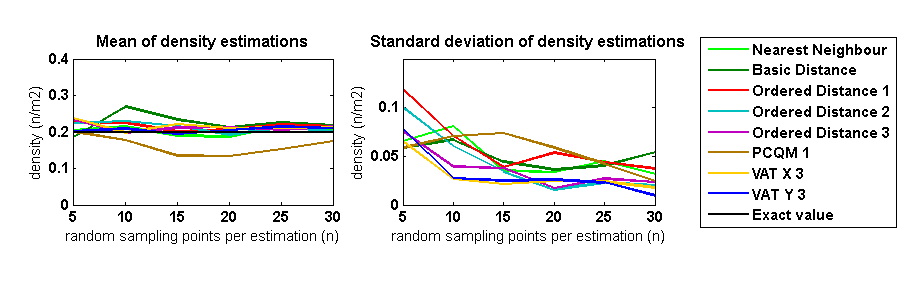


*Fig. 7)Means and standard deviations of tree density site a random pattern* *are plotted against the number of random sampling points using plotless sampling techniques. A repetition factor of 10 was used for the calculation.*

## 3.2 Estimates of tree density in a random pattern with density trend


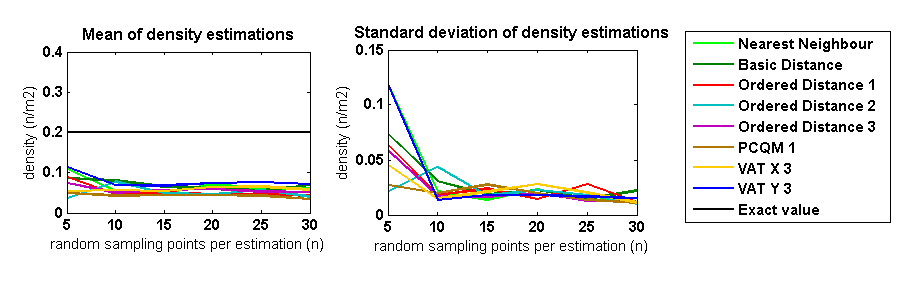


*Fig. 8)Means and standard deviations of tree density in a random pattern with trend are plotted against the number of random sampling points using plotless sampling techniques. A repetition factor of 10 was used for the calculation.*

## 3.3 Estimates of tree density in a regular pattern


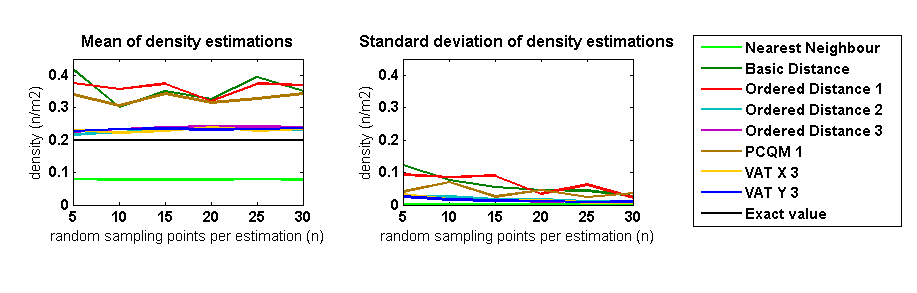


*Fig. 9)Means and standard deviations of tree density in a regular pattern* *are plotted against the number of random sampling points using plotless sampling techniques. A repetition factor of 10 was used for the calculation.*

## 3.4 Estimates of tree density in a regular pattern with density trend


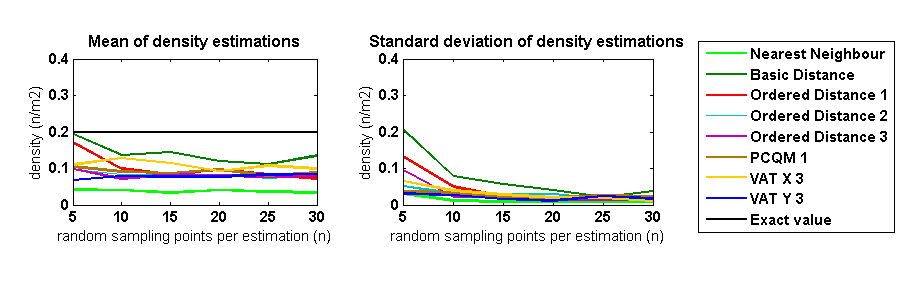


*Fig. 10)Means and standard deviations of tree density in a regular pattern with trend are plotted against the number of random sampling points using plotless sampling techniques. A repetition factor of 10 was used for the calculation.*

## 3.5 Estimates of tree density in an aggregated pattern


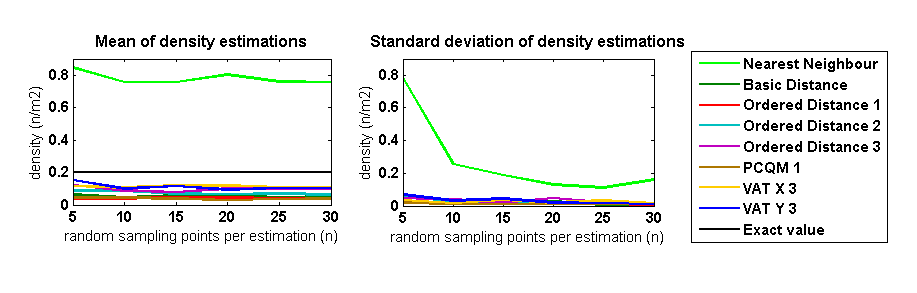


*Fig. 11)Means and standard deviations of tree density in an aggregated pattern are plotted against the number of random sampling points using plotless sampling techniques. A repetition factor of 10 was used for the calculation.*

## 3.6 Estimates of tree density in an aggregated pattern with density trend


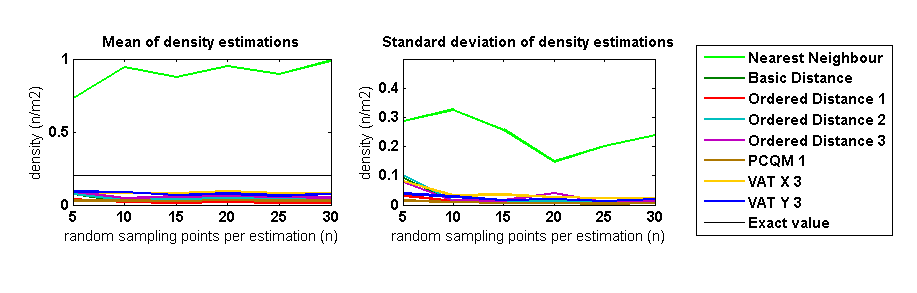


*Fig. 12)Means and standard deviations of tree density in an aggregated pattern with trend* *are plotted against the number of random sampling points using plotless sampling techniques. A repetition factor of 10 was used for the calculation.*

# References cited

1. Cottam G, Curtis JT (1956) The Use of Distance Measures in Phytosociological Sampling. Ecology 37: 451-460.

2. Cintrón G, Schaeffer Novelli Y (1984) Methods for studying mangrove structure. In: Snedaker SC, Snedaker JG, editors. The mangrove ecosystem: research methods: United Nations Educational, Scientific and Cultural Organization.
