# Supplementary material for: An Evaluation of Plotless Sampling Using Vegetation Simulations and Field Data from a Mangrove Forest
Source: PLoS One. 2013 Jun 27;8(6):e67201. doi: 10.1371/journal.pone.0067201 (PMC3695089; doi:10.1371/journal.pone.0067201)
Supplement: File S3 — Effect of density itself on mean density estimations. The vegetation patterns used in this study had a density of 0.2 trees/m2. To test the influence of the choice of this density on the results, we also varied the density between 0.05 and 1 trees/m2. The results found are presented in 6 tables, one for each vegetation pattern. (DOC) [file pone.0067201.s003.doc]

**Effect of density itself on mean density estimations**

# Table of contents

[0. Table of contents 1](#__RefHeading___Toc357599942)

[1. Introduction 1](#__RefHeading___Toc357599946)

[2. Results 1](#__RefHeading___Toc357599951)

[2.1 Random pattern 1](#__RefHeading___Toc357599957)

[2.2 Random pattern with trend 2](#__RefHeading___Toc357599958)

[2.3 Semi-regular pattern 2](#__RefHeading___Toc357599966)

[2.4 Semi-regular pattern with trend 3](#__RefHeading___Toc357599967)

[2.5 Aggregated pattern 3](#__RefHeading___Toc357599968)

[2.6 Aggregated pattern with trend 4](#__RefHeading___Toc357599969)

[3. References cited 5](#__RefHeading___Toc357599970)

# Introduction

In our study we have assessed the accuracy of density estimations using plotless sampling under different vegetation patterns. While testing, we kept the density of these patterns at 0.2 trees/m^2^. The question is if relative errors will change if this density would vary. In our 4 field sites, densities were found between 0.08 and 1.22 trees/m^2^. To see the effect of density variation we have varied the density of each vegetation patterns between 0.05 and 1 trees/m^2^. For the aggregated patterns, the cluster size have been kept constant, which influences the aggregation index R. Therefore, the aggregation indices are given for each pattern.

# Results

From tables 2 to 9 it can be seen that the accuracy of plotless sampling on average becomes higher when the density is higher. Only when a trend is present, the accuracy becomes less with higher densities, probably because the appearance of the trend also becomes stronger. Under clustered patterns, higher density seems to have a stronger effect on the error, because the degree of aggregation also increases with lower density. The question remains if the sizes of tree clusters change when tree density changes in the field.

The Variable Area Transect Method (VAT) seems to perform best under varying tree density. These results ask for more elaboration and research. As concise conclusions upon the influencing factors cannot be made for now, these results are shown in the supplementary material, hoping these can serve as a basis for more and more specific research.

## 2.1 Random pattern

*Table 1) Mean estimations, half width of 95% Confidence Intervals and relative mean errors using plotless sampling methods for random patterns with varying densities.*

| Mean of density estimations and half widths of 95% confidence intervals 930 sampling points, 10 replicates) | **Random pattern** | | | | | | | | |
| --- | --- | --- | --- | --- | --- | --- | --- | --- | --- |
|  | low density (R = 1.04) | | | medium density (R = 1.03) | | | high density (R = 1.00) | | |
|  | mean (n/m2) | half 95 CI (n/m2) | relative mean error (%) | mean (n/m2) | half 95 CI (n/m2) | relative mean error (%) | mean (n/m2) | half 95 CI (n/m2) | relative mean error (%) |
| **exact density** | **0.05** |  |  | **0.2** |  |  | **1** |  |  |
| **Nearest Neighbour** | 0.058 | 0.017 | **17%** | 0.22 | 0.08 | **12%** | 1.18 | 0.58 | **18%** |
| **Basic Distance** | 0.056 | 0.021 | **13%** | 0.22 | 0.09 | **9%** | 0.98 | 0.26 | **-2%** |
| **Ordered Distance 1** | 0.053 | 0.015 | **6%** | 0.21 | 0.04 | **7%** | 1.00 | 0.55 | **0%** |
| **Ordered Distance 2** | 0.051 | 0.010 | **3%** | 0.22 | 0.05 | **8%** | 1.09 | 0.25 | **9%** |
| **Ordered Distance 3** | 0.049 | 0.007 | **-1%** | 0.20 | 0.03 | **-2%** | 1.00 | 0.22 | **0%** |
| **PCQM 1** | 0.029 | 0.008 | **-41%** | 0.17 | 0.04 | **-16%** | 0.89 | 0.41 | **-11%** |
| **PCQM 2** | 0.035 | 0.011 | **-31%** | 0.16 | 0.05 | **-18%** | 0.68 | 0.44 | **-32%** |
| **PCQM 3** | 0.034 | 0.011 | **-32%** | 0.13 | 0.06 | **-34%** | 0.78 | 0.44 | **-22%** |
| **VAT X 3** | 0.059 | 0.011 | **19%** | 0.23 | 0.04 | **14%** | 0.93 | 0.15 | **-7%** |
| **VAT Y 3** | 0.064 | 0.009 | **28%** | 0.20 | 0.04 | **1%** | 0.98 | 0.13 | **-2%** |

## 2.2 Random pattern with trend

*Table 2) Mean estimations, half width of 95% Confidence Intervals and relative mean errors using plotless sampling methods for random patterns with trend and* varying densities.

| Mean of density estimations and half widths of 95% confidence intervals 930 sampling points, 10 replicates) | Random pattern with trend | | | | | | | | |
| --- | --- | --- | --- | --- | --- | --- | --- | --- | --- |
|  | low density (R = 0.83) | | | medium density (R = 0.90) | | | high density (R = 0.87) | | |
|  | mean (n/m2) | half 95 CI (n/m2) | relative mean error (%) | mean (n/m2) | half 95 CI (n/m2) | relative mean error (%) | mean (n/m2) | half 95 CI (n/m2) | relative mean error (%) |
| **exact density** | **0.05** |  |  | **0.2** |  |  | **1** |  |  |
| **Nearest Neighbour** | 0.018 | 0.015 | **-63%** | 0.09 | 0.05 | **-54%** | 0.47 | 0.24 | **-53%** |
| **Basic Distance** | 0.022 | 0.007 | **-56%** | 0.07 | 0.04 | **-63%** | 0.36 | 0.35 | **-64%** |
| **Ordered Distance 1** | 0.017 | 0.007 | **-65%** | 0.05 | 0.03 | **-75%** | 0.29 | 0.16 | **-71%** |
| **Ordered Distance 2** | 0.018 | 0.007 | **-64%** | 0.06 | 0.04 | **-71%** | 0.40 | 0.26 | **-60%** |
| **Ordered Distance 3** | 0.019 | 0.006 | **-62%** | 0.06 | 0.01 | **-71%** | 0.37 | 0.14 | **-63%** |
| **PCQM 1** | 0.021 | 0.009 | **-58%** | 0.05 | 0.03 | **-77%** | 0.24 | 0.15 | **-76%** |
| **PCQM 2** | 0.017 | 0.004 | **-67%** | 0.05 | 0.02 | **-77%** | 0.22 | 0.17 | **-78%** |
| **PCQM 3** | 0.017 | 0.007 | **-65%** | 0.04 | 0.03 | **-79%** | 0.21 | 0.09 | **-79%** |
| **VAT X 3** | 0.046 | 0.010 | **-8%** | 0.09 | 0.03 | **-53%** | 0.30 | 0.12 | **-70%** |
| **VAT Y 3** | 0.048 | 0.016 | **-4%** | 0.09 | 0.03 | **-55%** | 0.28 | 0.11 | **-72%** |

## 2.3 Semi-regular pattern

*Table 3) Mean estimations, half width of 95% Confidence Intervals and relative mean errors using plotless sampling methods for semi-regular patterns with varying densities.*

| Mean of density estimations and half widths of 95% confidence intervals 930 sampling points, 10 replicates) | Semi-regular pattern | | | | | | | | |
| --- | --- | --- | --- | --- | --- | --- | --- | --- | --- |
|  | low density (R = 1.87) | | | medium density (R = 1.89) | | | high density (R = 1.90) | | |
|  | mean (n/m2) | half 95 CI (n/m2) | relative mean error (%) | mean (n/m2) | half 95 CI (n/m2) | relative mean error (%) | mean (n/m2) | half 95 CI (n/m2) | relative mean error (%) |
| **exact density** | **0.05** |  |  | **0.2** |  |  | **1** |  |  |
| **Nearest Neighbour** | 0.019 | 0.001 | **-62%** | 0.08 | 0.00 | **-60%** | 0.39 | 0.01 | **-61%** |
| **Basic Distance** | 0.087 | 0.016 | **74%** | 0.34 | 0.08 | **72%** | 1.78 | 0.56 | **78%** |
| **Ordered Distance 1** | 0.093 | 0.023 | **86%** | 0.36 | 0.08 | **81%** | 1.88 | 0.50 | **88%** |
| **Ordered Distance 2** | 0.060 | 0.010 | **21%** | 0.24 | 0.01 | **19%** | 1.23 | 0.11 | **23%** |
| **Ordered Distance 3** | 0.058 | 0.006 | **17%** | 0.24 | 0.02 | **18%** | 1.21 | 0.11 | **21%** |
| **PCQM 1** | 0.076 | 0.018 | **52%** | 0.33 | 0.07 | **66%** | 1.75 | 0.28 | **75%** |
| **PCQM 2** | 0.043 | 0.018 | **-14%** | 0.18 | 0.06 | **-8%** | 1.11 | 0.38 | **11%** |
| **PCQM 3** | 0.036 | 0.020 | **-27%** | 0.17 | 0.11 | **-17%** | 0.96 | 0.47 | **-4%** |
| **VAT X 3** | 0.060 | 0.010 | **21%** | 0.24 | 0.02 | **22%** | 1.24 | 0.11 | **24%** |
| **VAT Y 3** | 0.063 | 0.010 | **26%** | 0.24 | 0.02 | **19%** | 1.19 | 0.13 | **19%** |

## 2.4 Semi-regular pattern with trend

*Table 4) Mean estimations, half width of 95% Confidence Intervals and relative mean errors using plotless sampling methods for semi-regular patterns with trend and varying densities.*

| Mean of density estimations and half widths of 95% confidence intervals 930 sampling points, 10 replicates) | Semi-regular pattern with trend | | | | | | | | |
| --- | --- | --- | --- | --- | --- | --- | --- | --- | --- |
|  | low density (R = 1.39) | | | medium density (R = 1.46) | | | high density (R = 1.47) | | |
|  | mean (n/m2) | half 95 CI (n/m2) | relative mean error (%) | mean (n/m2) | half 95 CI (n/m2) | relative mean error (%) | mean (n/m2) | half 95 CI (n/m2) | relative mean error (%) |
| **exact density** | **0.05** |  |  | **0.2** |  |  | **1** |  |  |
| **Nearest Neighbour** | 0.012 | 0.006 | **-77%** | 0.04 | 0.02 | **-78%** | 0.18 | 0.06 | **-82%** |
| **Basic Distance** | 0.035 | 0.009 | **-30%** | 0.14 | 0.10 | **-28%** | 0.64 | 0.29 | **-36%** |
| **Ordered Distance 1** | 0.028 | 0.018 | **-43%** | 0.13 | 0.07 | **-37%** | 0.62 | 0.69 | **-38%** |
| **Ordered Distance 2** | 0.021 | 0.006 | **-58%** | 0.09 | 0.02 | **-55%** | 0.40 | 0.17 | **-60%** |
| **Ordered Distance 3** | 0.024 | 0.008 | **-51%** | 0.09 | 0.01 | **-53%** | 0.43 | 0.14 | **-57%** |
| **PCQM 1** | 0.034 | 0.016 | **-33%** | 0.12 | 0.06 | **-40%** | 0.56 | 0.18 | **-44%** |
| **PCQM 2** | 0.022 | 0.009 | **-56%** | 0.08 | 0.02 | **-61%** | 0.29 | 0.12 | **-71%** |
| **PCQM 3** | 0.021 | 0.012 | **-59%** | 0.07 | 0.04 | **-64%** | 0.30 | 0.11 | **-70%** |
| **VAT X 3** | 0.064 | 0.028 | **28%** | 0.11 | 0.03 | **-46%** | 0.35 | 0.13 | **-65%** |
| **VAT Y 3** | 0.042 | 0.011 | **-16%** | 0.11 | 0.05 | **-47%** | 0.40 | 0.16 | **-60%** |

## 2.5 Aggregated pattern

*Table 5) Mean estimations, half width of 95% Confidence Intervals and relative mean errors using plotless sampling methods for aggregated patterns with varying densities.*

| Mean of density estimations and half widths of 95% confidence intervals 930 sampling points, 10 replicates) | Aggregated pattern | | | | | | | | |
| --- | --- | --- | --- | --- | --- | --- | --- | --- | --- |
|  | low density (R = 0.23) | | | medium density (R = 0.45) | | | high density (R = 0.84) | | |
|  | mean (n/m2) | half 95 CI (n/m2) | relative mean error (%) | mean (n/m2) | half 95 CI (n/m2) | relative mean error (%) | mean (n/m2) | half 95 CI (n/m2) | relative mean error (%) |
| **exact density** | **0.05** |  |  | **0.2** |  |  | **1** |  |  |
| **Nearest Neighbour** | 0.680 | 0.229 | **1260%** | 0.85 | 0.37 | **327%** | 1.11 | 0.45 | **11%** |
| **Basic Distance** | 0.011 | 0.006 | **-78%** | 0.05 | 0.03 | **-74%** | 0.63 | 0.24 | **-37%** |
| **Ordered Distance 1** | 0.009 | 0.007 | **-81%** | 0.03 | 0.02 | **-83%** | 0.62 | 0.20 | **-38%** |
| **Ordered Distance 2** | 0.015 | 0.006 | **-71%** | 0.08 | 0.04 | **-61%** | 0.74 | 0.31 | **-26%** |
| **Ordered Distance 3** | 0.019 | 0.005 | **-62%** | 0.09 | 0.03 | **-53%** | 0.74 | 0.30 | **-26%** |
| **PCQM 1** | 0.007 | 0.002 | **-85%** | 0.04 | 0.02 | **-81%** | 0.39 | 0.23 | **-61%** |
| **PCQM 2** | 0.013 | 0.003 | **-74%** | 0.06 | 0.01 | **-70%** | 0.41 | 0.36 | **-59%** |
| **PCQM 3** | 0.018 | 0.002 | **-64%** | 0.08 | 0.03 | **-60%** | 0.43 | 0.34 | **-57%** |
| **VAT X 3** | 0.044 | 0.017 | **-12%** | 0.12 | 0.06 | **-40%** | 0.85 | 0.29 | **-15%** |
| **VAT Y 3** | 0.047 | 0.011 | **-6%** | 0.12 | 0.02 | **-39%** | 0.83 | 0.30 | **-17%** |

## 2.6 Aggregated pattern with trend

*Table 6) Mean estimations, half width of 95% Confidence Intervals and relative mean errors using plotless sampling methods for aggregated patterns with trend and varying densities.*

| Mean of density estimations and half widths of 95% confidence intervals 930 sampling points, 10 replicates) | Aggregated pattern with trend | | | | | | | | |
| --- | --- | --- | --- | --- | --- | --- | --- | --- | --- |
|  | low density (R = 0.23) | | | medium density (R = 0.42) | | | high density (R = 0.75) | | |
|  | mean (n/m2) | half 95 CI (n/m2) | relative mean error (%) | mean (n/m2) | half 95 CI (n/m2) | relative mean error (%) | mean (n/m2) | half 95 CI (n/m2) | relative mean error (%) |
| **exact density** | 0.05 |  |  | **0.2** |  |  | **1** |  |  |
| **Nearest Neighbour** | 0.804 | 0.421 | **1508%** | 0.81 | 0.24 | **307%** | 1.03 | 0.44 | **3%** |
| **Basic Distance** | 0.005 | 0.002 | **-90%** | 0.02 | 0.02 | **-88%** | 0.29 | 0.20 | **-71%** |
| **Ordered Distance 1** | 0.004 | 0.002 | **-91%** | 0.02 | 0.02 | **-91%** | 0.19 | 0.08 | **-81%** |
| **Ordered Distance 2** | 0.008 | 0.001 | **-85%** | 0.03 | 0.02 | **-83%** | 0.31 | 0.18 | **-69%** |
| **Ordered Distance 3** | 0.010 | 0.002 | **-79%** | 0.05 | 0.04 | **-75%** | 0.37 | 0.11 | **-63%** |
| **PCQM 1** | 0.004 | 0.001 | **-91%** | 0.03 | 0.02 | **-85%** | 0.18 | 0.11 | **-82%** |
| **PCQM 2** | 0.008 | 0.001 | **-84%** | 0.05 | 0.02 | **-76%** | 0.19 | 0.14 | **-81%** |
| **PCQM 3** | 0.011 | 0.002 | **-77%** | 0.06 | 0.02 | **-70%** | 0.32 | 0.21 | **-68%** |
| **VAT X 3** | 0.042 | 0.010 | **-17%** | 0.09 | 0.03 | **-54%** | 0.42 | 0.27 | **-58%** |
| **VAT Y 3** | 0.030 | 0.006 | **-40%** | 0.08 | 0.03 | **-60%** | 0.36 | 0.23 | **-64%** |

# References cited

1. Clark PJ, Evans FC (1954) Distance to Nearest Neighbor as a Measure of Spatial Relationships in Populations. Ecology 35: 445-453.
